# Interaction between floral rewards and floral symmetry shapes diversification dynamics in Amazonian trees

**DOI:** 10.1111/nph.70623

**Published:** 2025-10-08

**Authors:** Diego Graciano, Gustavo Burin, Sandra Maria Carmello‐Guerreiro, Elisabeth Dantas Tölke

**Affiliations:** ^1^ Departamento de Biologia Vegetal, Instituto de Biologia Universidade Estadual de Campinas – UNICAMP CEP 13083‐970 Campinas São Paulo Brazil; ^2^ Department of Biosciences Durham University South Road Durham DH1 3LE UK; ^3^ Department of Biological and Environmental Sciences University of Gothenburg Medicinaregatan 7B Gothenburg 413 90 Sweden; ^4^ Departamento de Biologia, Centro de Ciências Biológicas e da Saúde Universidade Estadual da Paraíba CEP 58429‐500 Campina Grande Paraíba Brazil

**Keywords:** floral symmetry, Lecythidaceae, nectar, phylogenetic comparative methods, pollen, pollination, species diversification

## Abstract

- Floral zygomorphy, or monosymmetry, is thought to have a positive effect on the diversification rates of angiosperms, but its true impact is still an open topic. Given the controversy surrounding this matter, our study evaluates whether rewards such as nectar and pollen produced by floral structures evolve in correlation with floral symmetry and its effects on diversification.
- Using the family Lecythidaceae, we characterized the floral structures that produce different rewards for pollinators and assessed whether these rewards evolve correlated with the varying levels of floral symmetry observed in the subfamily Lecythidoideae. We also used trait‐dependent diversification to assess whether this trait correlation affects lineage diversification rates.
- Floral rewards are produced by structures that are morphological modifications of the androecium, resulting in unusual floral nectaries and leading to a gradual sterilization of the male function in the flowers. The kinds of rewards evolve in a correlated fashion with the varying levels of floral symmetry observed in the subfamily, with nectar production being most likely associated with strongly monosymmetric floral forms. Lastly, our results suggest that this combination of trait states, nectar, and monosymmetry, is loosely associated with increased diversification in Lecythidoideae.
- We propose that these patterns might be related to the degree of specialization of pollinators attracted by the different types of flowers and rewards, and to the complex interaction between biotic and abiotic factors; however, this is still open to debate. The evaluation of correlated floral traits, especially symmetry and nectar production, is essential for a better understanding of the diversification dynamics of angiosperm lineages.

## Introduction

Intrinsic traits of organisms play a fundamental role in the dynamics of lineage diversification (O'Meara *et al*., 2016; Vamosi *et al*., 2018; Anderson *et al*., 2023; Helmstetter *et al*., 2023). Understanding how these traits can positively or negatively affect diversification rates is a central question in angiosperm macroevolution (for a review, see Sauquet & Magallón, 2018). Many traits influence the way plants interact with the biotic and abiotic environments, thus affecting the biology of the species (Sanderson & Donoghue, 1994). Therefore, these traits are expected to influence species evolution over time. Intrinsic traits that can positively and significantly influence diversification rates of a clade are considered key innovations (Hodges & Arnold, 1995; Hunter, 1998). In angiosperms, many key innovations are associated with flowers due to their functional role in speciation (Stebbins, 1970; Vamosi *et al*., 2018). The presence of flowers correlates with greater diversification in angiosperms, driven by specific floral character states (Vamosi & Vamosi, 2011). Thus, some modifications and innovations in floral shape appear to have favored the diversification of this group of plants (Endress, 2011).

Floral zygomorphy, also known as floral monosymmetry, is one of the intrinsic traits with the greatest potential to drive diversification rates in angiosperm lineages (Vamosi *et al*., 2018). The presence of bilaterally symmetric flowers can increase pollination specificity by facilitating the process of pollinator approach and landing on the flower, as well as allowing for more precise pollen deposition on the pollinator's body (Citerne *et al*., 2010). Increasing evidence supports the notion that floral monosymmetry enhances precise pollen placement on different functional groups of pollinators (Stebbins, 1951; Neal *et al*., 1998; Fenster *et al*., 2009; Culbert & Forrest, 2016; Jirgal & Ohashi, 2023), particularly in tropical environments (Stewart *et al*., 2022). If monosymmetry favors pollination specialization, adaptive divergence and reproductive isolation are more likely to occur, leading to higher speciation rates in lineages with this trait (Sargent, 2004; Citerne *et al*., 2010; Anderson *et al*., 2023). Indeed, monosymmetric lineages tend to exhibit greater species diversity than their polysymmetric sister lineages (Sargent, 2004), and the presence of monosymmetry significantly contributes to the imbalance of angiosperm clades (Vamosi & Vamosi, 2011). Contrary to expectations, however, monosymmetry can also lead to increased extinction rates in the long term due to the risks associated with the loss of specialized pollinators (Knight *et al*., 2005; Yoder *et al*., 2020). Therefore, its overall impact on net diversification rates (speciation minus extinction) remains debated. For instance, in Proteaceae, floral monosymmetry contributed to increased speciation and extinction rates, but did not significantly affect the net diversification rate of the family (Reyes *et al*., 2015). This inconsistency has been key to arguments that traits that are thought to have consistent effects on diversification actually end up having inconsistent effects (Anderson *et al*., 2023).

Although studies have examined the evolutionary patterns of floral symmetry, none have quantified the relative importance of differential diversification resulting from the combination of this trait with the kind of floral reward, which may promote functional interactions (e.g. Dellinger *et al*., 2021). Because these studies consider the evolution of symmetry in isolation, they assume that traits evolve independently of each other. However, clade diversification may be associated with trait interdependence, either through direct or through indirect correlation between individual traits (phenotypic integration) (Donoghue & Sanderson, 2015; Sauquet & Magallón, 2018; Vamosi *et al*., 2018; Zenil‐Ferguson *et al*., 2019). In these cases, natural selection may act simultaneously on multiple combinations of floral traits (Fenster *et al*., 2015). A recent analysis indicates that diversification rates in angiosperms are influenced by hidden character states related to floral symmetry (Wang *et al*., 2023), suggesting that the effect of symmetry on diversification may depend on interactions with other traits (Anderson *et al*., 2023), such as nectar and food‐pollen production.

**Fig. 1:**
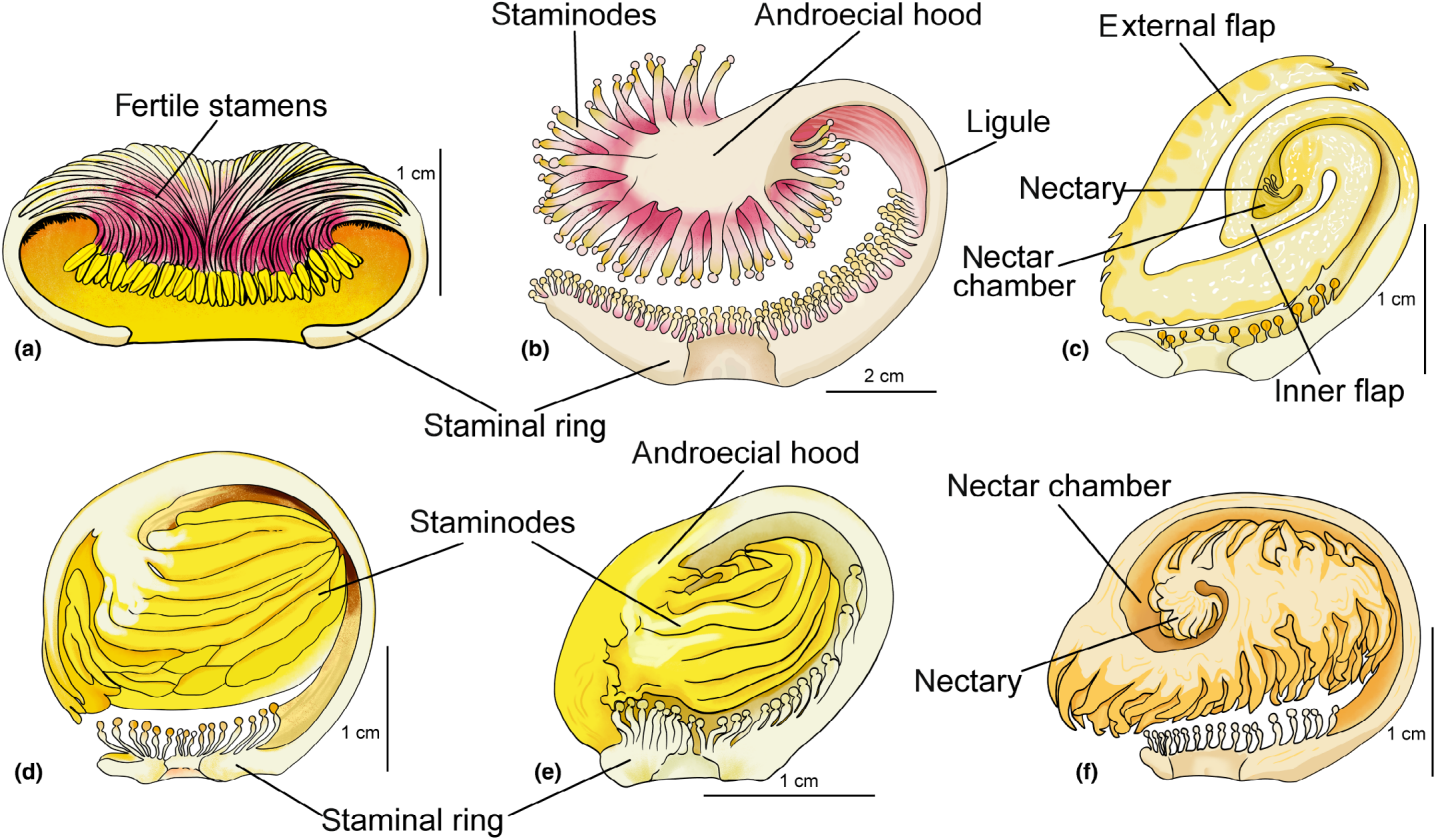
Structure of the androecium in Lecythidoideae. Diagrams of longitudinal sections showing the androecium of flowers at anthesis. (a) Example of *Grias* and *Gustavia*. The androecium is polysymmetric, with the stamens inserted at the edge of a staminal ring or tube. (b) *Couroupita guianensis*. The stamens form a staminal ring around the gynoecium, with a unilateral extension called a ligule and an androecial hood containing staminodes that produce foraging pollen. (c) Example of *Couratari*. The ligule curves inward, forming an inner flap, and the hood curves outward, forming an outer flap. (d) *Bertholletia excelsa*. The distal region of the ligule contains staminodes; this region is defined as the androecial hood by Mori & Prance (1990). (e) *Lecythis prancei*. Note that the staminodes are curved inward into the androecial hood. (f) Example of *Eschweilera*. The ligule curves over the staminal ring and gynoecium, forming a spiral androecial hood with nectar‐producing staminodes. (a–f) Illustrations redrawn from Prance & Mori (1979).

Tropical regions are home to the most species‐rich biomes on the planet, providing a variety of biotic and abiotic interactions (Brown, 2014). These interactions exert selective pressures that influence speciation and extinction rates and shape phenotypic diversity (Donoghue, 2005; Sargent & Ackerly, 2008; Schemske *et al*., 2009). Within this scenario, Lecythidaceae *sensu lato* is a pantropical family of angiosperms comprising *c*. 355 species (Mori *et al*., 2017). Its greatest diversity is found in the Amazon rainforest, where it is the third most abundant family in terms of the number of individuals (ter Steege *et al*., 2013). A complex floral architecture makes it the family with the highest floral morphological disparity in the order Ericales (Chartier *et al*., 2021). This high disparity is partly due to floral symmetry, expressed in different ways in the subfamily Lecythidoideae, which is associated with attracting pollinators through different kinds of floral rewards. In this context, the reduction in the number of fertile stamens and the progressive establishment of monosymmetry in all sets of floral organs seem to be associated with a shift from fertile pollen to infertile pollen and finally to nectar as a reward (Mori *et al*., 2007; Huang *et al*., 2011; Mori *et al*., 2017). Alternatively, monosymmetry, manifested mainly in the androecium of Lecythidoideae, results from abaxial dominance in floral development and the evolution of an androecial hood (Tsou & Mori, 2007; Fig. 1). The evolution of a wide variety of androecial hoods is the result of selection for specialized and efficiently pollinated flowers and seems to explain the diversity of the subfamily (Tsou & Mori, 2007; Fig. 1). As a consequence, diversification analyses indicated that the Neotropical subfamily diversified at a relatively constant rate through time, with a slight acceleration in speciation rates toward the present (Vargas & Dick, 2020).

Therefore, the subfamily Lecythidoideae offers an excellent opportunity to investigate the evolutionary dynamics and the influence of changes in the kind of floral rewards correlated with floral symmetry on diversification. Here, we (1) morphoanatomically characterize the reward‐producing floral structures to understand their homology relationships and their role in attracting pollinators; (2) use 118 species of Lecythidaceae to examine the correlation between the evolution of floral rewards and the evolution of floral symmetry; and (3) evaluate the effect of the combination of changes in reward kind and floral symmetry on diversification rates in the subfamily Lecythidoideae based on multistate hidden state speciation and extinction models. The subfamily is undergoing a process of continuous diversification, the causes of which are fundamental to understanding the dynamics of speciation and biological diversification in the Amazon. In this context, we present evidence that the correlation between floral rewards and floral symmetry acts as a driver of speciation rates in a group of hyperdominant trees in Amazonian forests.

## Materials and Methods

### Morpho‐anatomical characterization of structures producing floral rewards and their coding

The species of Lecythidoideae were selected according to the most recent phylogeny for the subfamily (Vargas & Dick, 2020), also based on systematic reviews (Huang *et al*., 2015; Mori *et al*., 2015), to represent as much morphological and phylogenetic diversity as possible. We used flowers from the species listed in Supporting Information Table S1, representing the major clades of the Lecythidoideae phylogeny. Fresh flowers in pre‐anthesis and anthesis were fixed in Karnovsky's solution for 24 h (Karnovsky, 1965), dehydrated in an ascending ethanol series, and stored in 70% ethanol. Flower material was dehydrated in an ascending ethanol series and embedded in hydroxyethyl methacrylate (Historesin®; Leica Biosystems Nussloch GmbH, Nussloch, Germany) according to the manufacturer's recommendations. Transverse and longitudinal sections of 3–5 μm thickness were prepared using a rotary microtome (Microm HM340E; Thermo Fisher Scientific Inc., Waltham, MA, USA). Slides were stained with 0.05% toluidine blue in citrate buffer (pH 4.5) (O'Brien *et al*., 1964) and Nile red fluorochromes for lipids (Greenspan *et al*., 1985) under a fluorescence U‐MNIGA3 filter (excitation: BP 530 nm; emission: 620 nm), and autofluorescence under a U‐MWU2 filter (excitation: BP 340 nm; emission: 460 nm) (Cardoso‐Gustavson *et al*., 2018), and finally mounted in Entellan® resin (Merck KGaA, Darmstadt, Germany) or temporarily in water. Images were captured using a digital camera (Olympus DP71) attached to a light microscope (Olympus BX51; Olympus Optical Co. Ltd, Japan). For scanning electron microscopy (SEM) analysis, pre‐anthesis and anthesis flowers were fixed and dehydrated as described above and stored in 70% ethanol. They were then dehydrated in an ascending ethanol series, dried using the CO_2_ critical point method in a CPD‐030 apparatus, mounted, and sputter‐coated with colloidal gold. Observations and images were obtained using a Jeol JSM 5800 LV (JEOL Ltd, Tokyo, Japan) scanning electron microscope at 10 kV with an attached digital camera, with a working distance of 14 mm. Unedited SEM images are available in Dataset S1.

Information on the floral rewards in Lecythidoideae was obtained from monographs (Mori *et al*., 1978; Prance & Mori, 1979; Mori & Boeke, 1987; Mori *et al*., 1990), supplemented by data from ‘The Lecythidaceae Pages’ (https://sweetgum.nybg.org/lp/index.php; Mori *et al*., 2010). These sources provided access to detailed studies on the pollination biology of Lecythidoideae species, allowing us to accurately determine the kind of reward.

### Coding for floral symmetry

In Lecythidoideae, floral evolution exhibits three distinct levels of complexity with respect to floral symmetry. The first genera to diverge, *Grias* L. and *Gustavia* L., have polysymmetric flowers, corresponding to the first level, as they do not exhibit abaxial dominance in floral development (Fig. 1a). In some genera, such as *Couroupita* Aubl. and *Couratari* Aubl., monosymmetry is established by the expression of abaxial dominance in floral development and the emergence of an androecial hood affecting only the perianth and androecium, characterizing the second level (Fig. 1b,c). At the final level, abaxial dominance is expressed in floral development, and the gynoecium is also affected. In addition, the prolonged meristematic activity of the staminal ring meristem promotes hood development and influences gynoecium monosymmetry (Fig. 1d–f). The above information on floral symmetry was extracted from Tsou & Mori (2007), who examined the floral development of representatives from all genera of Lecythidoideae using scanning electron microscopy analyses. We classified flowers of species in the first level as ‘polysymmetric’, those in the second level as ‘partially monosymmetric’ due to gynoecium polysymmetry, and finally those in the third level as ‘strongly monosymmetric’, where bilateral flower symmetry affects all floral organs.

### Ancestral state estimation and correlated evolution between floral rewards and symmetry

To evaluate the relationship between the kind of floral rewards and floral symmetry in a phylogenetic framework, we used the best scoring and time‐calibrated ML phylogenetic tree constructed with 16 molecular markers for the subfamily Lecythidoideae. This tree, obtained from the study by Vargas & Dick (2020), includes 118 taxa, of which eight belong to the outgroup. We performed ancestral state estimations for both traits. First, we estimated the ancestral states of the characters across the phylogeny using different transition rate models (ARD), symmetric transitions (SYM), and equal rates (ER) through the *ace* function (ape 5.0; Paradis & Schliep, 2019). The ER model was preferred for the reward kind trait (ER AICc: 60.73, SYM AICc: 64.23, ARD AICc: 68.31). The ER model was also preferred for the floral symmetry trait (ER AICc: 54.15, SYM AICc: 58.15, ARD AICc: 61.61). We then performed stochastic character mapping under the ER model for both traits using *make.simmap* with 200 simulations (phytools; Revell, 2012). All analyses were performed using R v.4.2.3 (R Core Team, 2019).

Next, we tested for correlated evolution between reward kind and floral symmetry using the reversible‐jump MCMC algorithm implemented in BayesTraits v.5 with both independent and dependent models of character evolution (Pagel & Meade, 2006). Marginal likelihoods were estimated for each model using the stepping‐stone method. Because this method allows only binary traits, we binarized the reward kind as 0 – pollen, 1 – nectar, and floral symmetry as 0 – polysymmetry, 1 – monosymmetry (Dataset S2). Finally, we used the log Bayes Factor (LBF) criterion established by Kass & Raftery (1995) for evidence of correlated evolution.

### Trait‐dependent diversification models

To evaluate the effect of the combination of floral rewards and floral symmetry on diversification dynamics in Lecythidoideae, we generated new character states based on a simple combination of the traits of interest: 0 – Pollen + Polysymmetry, 1 – Pollen + Monosymmetry, and 2 – Nectar + Monosymmetry. We used the already‐established hidden state speciation and extinction (HiSSE) model and the multistate HiSSE (MuHiSSE) model framework to estimate the state‐dependent diversification rates (Beaulieu & O'Meara, 2016). We fitted three models to our data: a null model for which the rates do not vary with the character state; the standard 3‐state MuSSE model; and a model in which the differences in rates are not associated with the mapped trait but instead are related to an unmapped, hidden state. All three models were parameterized using turnover rates and extinction fraction, following the documentation, to facilitate optimization. We opted not to fit the full MuHiSSE model (which includes hidden states to the standard MuSSE model) due to an unfavorable data‐to‐parameter ratio (J. Beaulieu, pers. comm.). We then compared models using their respective AICc and AICw values. Due to the nature of our trait, it could be possible that either one or the other component trait (reward or symmetry) is driving the diversification dynamics. Hence, we also fitted similar models to each of the component traits: a null model, with rates independent of the states; a standard BiSSE model (Pollen vs Nectar for ‘reward’ and Polysymmetry vs Monosymmetry for ‘symmetry’); and a HiSSE model, including one hidden state. The parameterization for these models follows the same rationale as the main analysis, and the models were also compared using their respective AICc and AICw.

### Generalized linear mixed models (GLMMs)

According to Helmstetter *et al*. (2023), the application of trait‐dependent diversification methods should be guided by several criteria: phylogenetic sampling of at least 25%, a frequency of the rarest state of at least 10% in the phylogeny, multiple (though not excessive) independent origins of derived states, and preferably, the use of phylogenies with > 300 terminals. Our dataset meets all of these recommendations except the last one, as the ingroup phylogeny includes only 110 terminals (Vargas & Dick, 2020). We acknowledge that the trait‐dependent diversification models can be seen as quite parameter‐rich for the size of our phylogeny, rendering the parameter estimates questionable at best. To provide some more information to the discussion regarding rate differences between states, we used generalized linear mixed models to test for differences in speciation rates at present between each of the three states. We used the speciation rates estimated at the tips of the phylogeny using CLaDS (Maliet *et al*., 2019; Maliet & Morlon, 2022) and grouped the species according to the three states of the composite trait. We set the sampling fraction to 0.43 and sampled 100 trees from the collection of trees derived from the data augmentation process. Fig. 6(a) shows the phylogeny obtained from Vargas & Dick (2020), including 110 species of Lecythidoideae, and includes both information on trait states and speciation rates.

To fit the mixed models to our data, we used the mcmcglmm package in R (Hadfield, 2010), which implements generalized linear mixed models in a Bayesian framework that allows us to include different sources of uncertainty in parameter estimation. We ran the chains for 5 million generations, discarding the first 50% as burn‐in, and sampled every 2500 generations, resulting in a posterior distribution of 1000 samples. We used inverse Wishart distributions as priors for the fixed and random effects and assessed the effect size of each state by analyzing the asymmetries of the posterior distributions in relation to zero (which would mean no effect).

## Results

### Floral rewards are produced by structures resulting from morphological modifications in the androecium

**Fig. 2:**
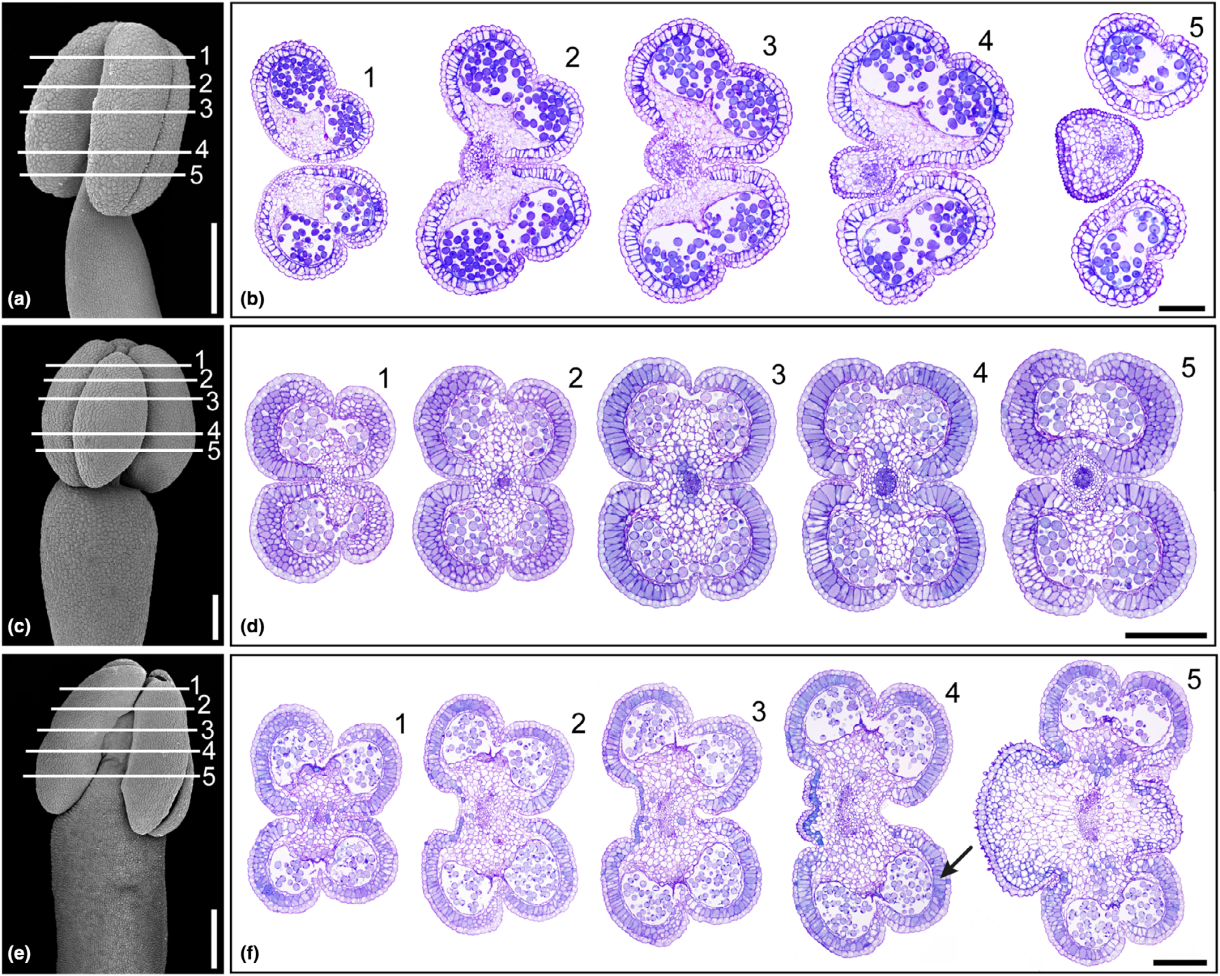
Androecium of Lecythidoideae. (a, b) *Grias neuberthii*. (a) Overview of the fertile stamen with the background removed; the horizontal lines and numbering correspond to those in (b). (b) Cross‐sections of the fertile anther. (c–f) *Couroupita guianensis*. (c) Overview of the fertile stamen from the staminal ring with the background removed; the horizontal lines and numbering correspond to those in (d). (d) Cross‐sections of the fertile anther. (e) Overview of the staminode from the androecial hood with the background removed; the horizontal lines and numbering correspond to those in (f). (f) Cross‐sections of the infertile anther. The arrow indicates a layer of poorly developed endothecium cells. Bars: (a, e) 500 μm; (b–d, f) 200 μm.

Lecythidoideae species provide three kinds of rewards to pollinators: fertile pollen, foraging pollen (infertile), and nectar. Fertile pollen is produced by stamens arranged in a staminal ring around the gynoecium, with bithecate and tetrasporangiate anthers (Figs 1, 2a–f). Fertile anthers often have phenolic idioblasts in the epidermis cells (e.g. *Couroupita* and *Couratari*) (Figs 2d, S1a,b). In monosymmetric species, there is a unilateral extension in the staminal ring forming a ligule (Fig. 1b–f). The distal region of the ligule features an androecial hood that curves over the floral receptacle where fertile stamens and/or staminodes are inserted (Fig. 1b–f). These may or may not contain anthers, which produce foraging pollen. In most taxa, fertile and sterile anthers have a layer of endothecium cells around the pollen sacs, with lignified wall thickenings, usually continuous in the ventral and dorsal regions of the connective (Fig. 2a–f). In other species, the anthers are dimorphic, and infertile anthers have endothecium cells with poorly developed wall thickenings (e.g. *Corythophora*; Fig. S1c–f).

**Fig. 3:**
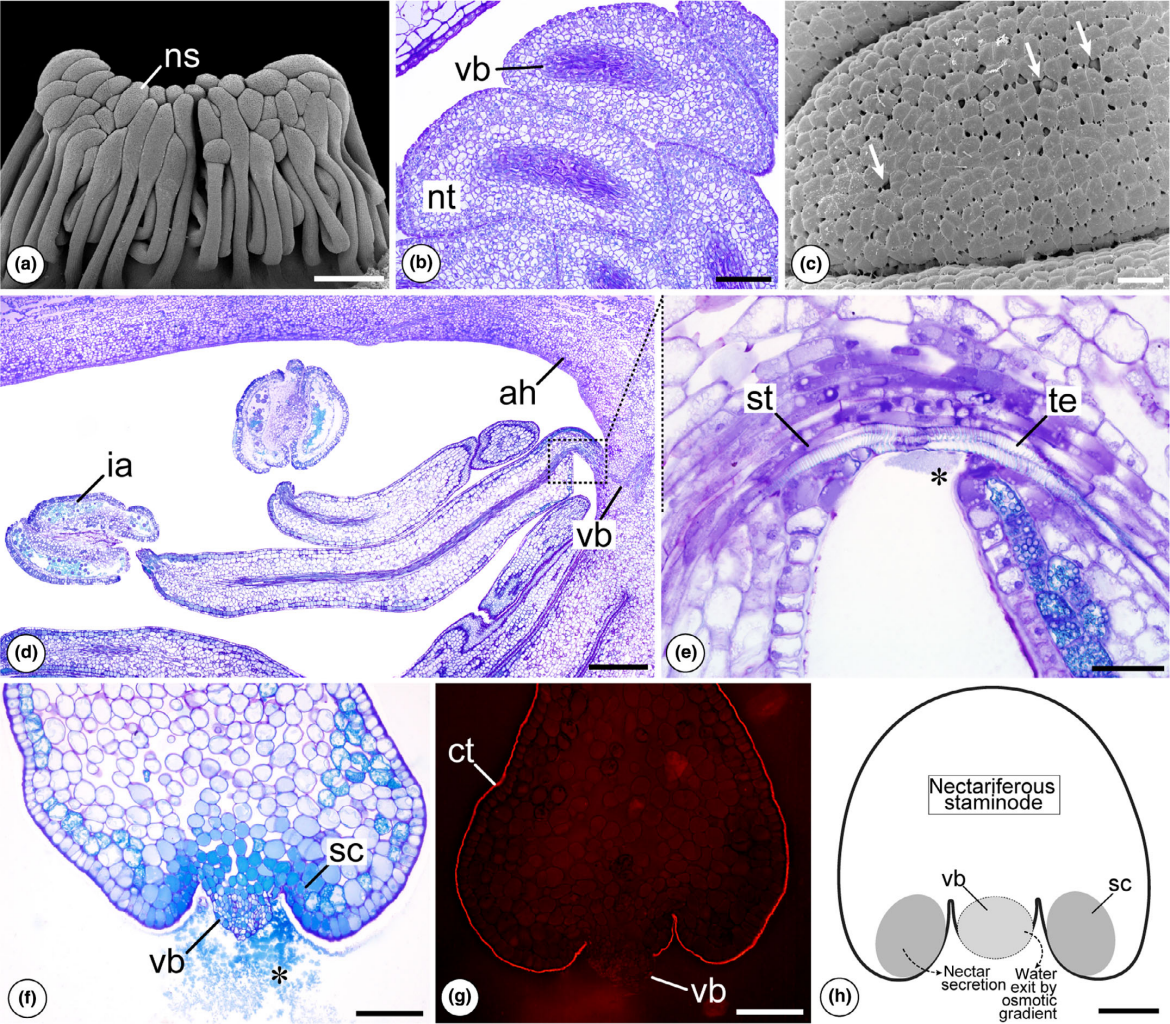
Structural characterization of nectaries in the species of the genus *Couratari* and *Lecythis* using scanning electron microscopy (SEM) and light microscopy. (a–g) *Couratari asterotricha*. (a) Overview of the staminode positioning in the nectar chamber with the background removed. Note the narrow proximal portion and the dilated distal portion. (b) Longitudinal section of the distal region of the staminodes. Note the nectariferous tissue and the amphicribal vascular bundles. (c) Surface of the distal region of the staminode. Arrows indicate the spacing of the epidermis. (d, e) *Lecythis poiteaui*. (d) Longitudinal section of the inner region of the androecial hood with nectar‐secreting staminodes. Note the course of the vascular bundle. (e) Detail of the region of insertion of the staminode in the androecial hood, showing the peripheral vascular bundle. * indicates nectar secretion. (f, g) *Lecythis prancei*. (f) Cross‐section of the base of the staminode showing the peripheral vascular bundle. * indicates nectar secretion. (g) Cross‐section of the base of the staminode under fluorescence after staining with Nile Red, showing the discontinuity of the cuticle in the region of the vascular bundle. (h) Hypothetical diagram of the base of the secretory staminode showing the pathway of nectar formation. ah, androecial hood; ct, cuticle; ia, infertile anther; ns, nectariferous staminode; nt, nectariferous tissue; sc, secretory cell; st, sieve tube; te, tracheal element; vb, vascular bundle. Bars: (a) 1 mm; (d) 500 μm; (b, f–h) 100 μm; (c, e) 50 μm.

The species that produce nectar exhibit complex and elaborate androecial hoods (Fig. 1c–f). Nectar‐producing staminodes might be present inside of a nectar chamber in hoods with a coil that curves toward the receptacle, from which a flap projects outward and folds toward the apex of the hood, as occurs in *Couratari* (Fig. 1c). In these cases, the expanded distal region of the staminodes is filled with nectariferous tissue and is responsible for the synthesis and release of nectar (Fig. 3a–c). In this region, the epidermis consists of noncuticular and nonjuxtaposed cells with conspicuous intercellular spaces through which the nectar is exuded (Fig. 3c). Another type of nectar production and secretion is observed in *Bertholletia excelsa* and some species of *Lecythis* and occurs at the junction of the staminodes with the androecial hood (Figs 3d, S2a–h). At the site of nectar release, an amphicribal vascular bundle follows a descending trajectory toward the staminode and approaches the epidermis of the hood (Fig. 3d). Part of the conducting cells, both xylem and phloem, is positioned just below the epidermis, while another part extends toward the staminode to carry out its vascularization (Fig. 3e). In the region of confluence between the conducting tissue and the external environment, the tracheal elements and sieve tubes are peripheral, and the continuity of the cuticle and epidermis cells in this region is absent (Fig. 3e–g). The primary cell wall of the conductive elements is the only barrier between these cells and the external environment. We propose that nectar is processed by cells adjacent to the vascular bundle at the base of the staminode and released into the cavity where the peripheral bundle is inserted. The water transported by the tracheal elements is released to the external environment by capillarity, probably due to an osmotic gradient generated by the presence of sugars secreted by the nectary (Fig. 3h).

**Fig. 4:**
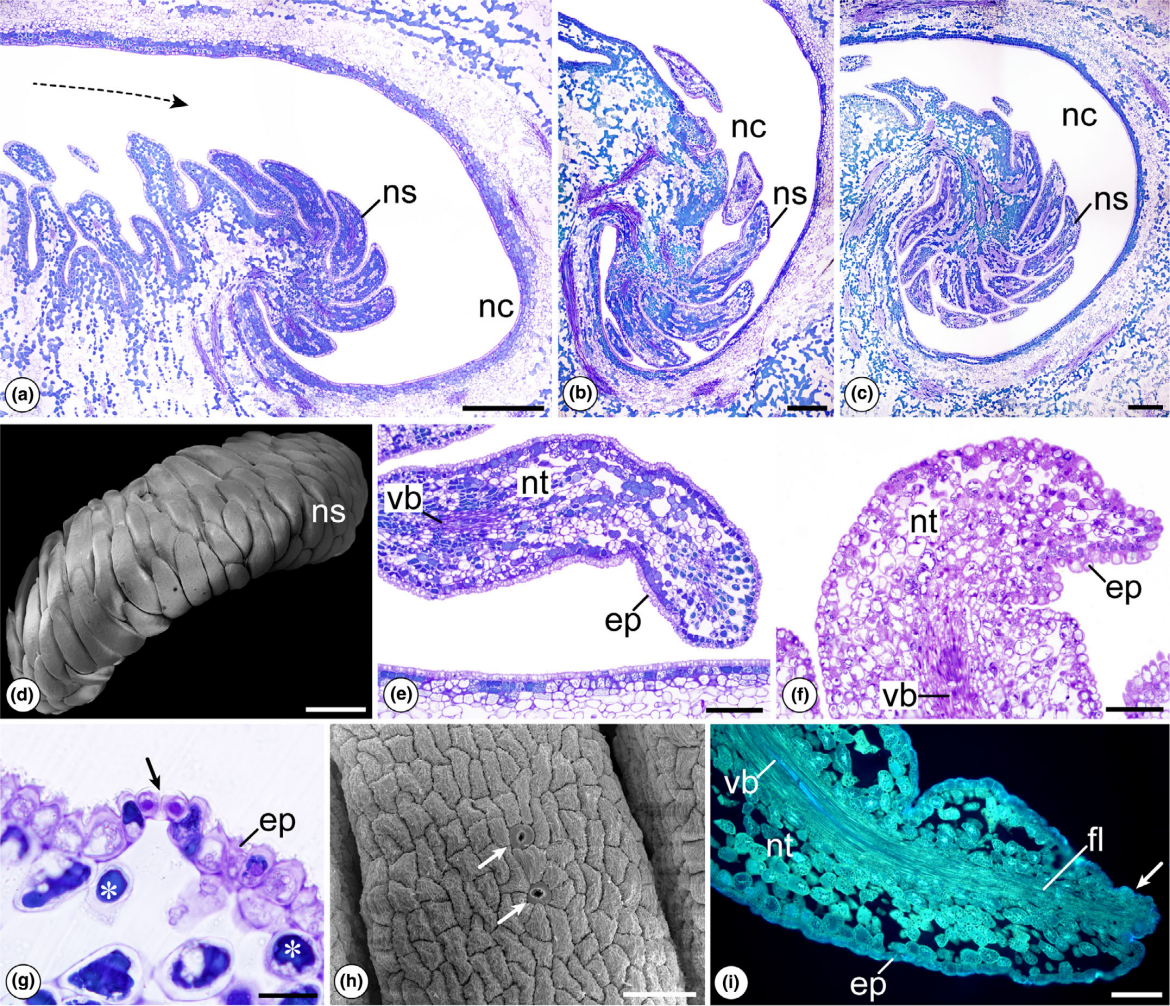
Structural characterization of nectaries in species of the genus *Eschweilera* using scanning electron microscopy (SEM) and light microscopy. (a) *Eschweilera coriacea*. Longitudinal section of the distal region of the androecial hood. The arrow indicates the narrow channel that serves as a passage for pollinators to the nectar chamber. (b) *Eschweilera atropetiolata*. Longitudinal section of the distal region of the androecial hood where the nectariferous staminodes are inserted. (c) *Eschweilera truncata*. Longitudinal section of the distal region of the androecial hood, where numerous nectariferous staminodes are inserted. (d, e) *Eschweilera atropetiolata*. Frontal view of the distal region of the androecial hood, showing the curved nectariferous staminodes with the background removed. Note the ‘U’‐shaped platform where the appendages are inserted. (e) Detail of the nectariferous staminode. Note the nectariferous tissue occupying the entire length of the staminode. (f) *Eschweilera wachenheimii*. Detail of the distal region of the nectariferous staminode. Note the heterogeneity of compounds present in cells of the nectariferous tissue. (g) *Eschweilera truncata*. Detail of the epidermal region of the nectariferous staminode. The arrow indicates secretory stomata. * indicates subepidermal parenchyma with phenolic idioblasts. (h) *Eschweilera atropetiolata*. Morphological surface of the nectariferous staminode. Arrows indicate secretory stomata. (i) *Eschweilera grandiflora*. Autofluorescence of the distal region of the nectariferous staminode. The arrow indicates the potentially reduced anther. ep, epidermis; fl, phloem; nc, nectar chamber; ns, nectariferous staminodes; nt, nectariferous tissue; vb, vascular bundle. Bars: (d) 1 mm; (a–c) 500 μm; (e) 200 μm; (f, i) 100 μm; (g, h) 50 μm.

The third type of nectary is found in the species of *Eschweilera*. The androecial hood curves inward and may form a single, double, or triple coil containing numerous nectariferous staminodes in the inner region of the spiral (Figs 1f, 4a–c). The staminodes are positioned on a ‘U‐shaped’ platform and curve toward the nectariferous cavity, where the nectar is secreted (Fig. 4d). These staminodes are filled with nectariferous tissue, and the nectar produced is exuded through stomata (Fig. 4e–h). A collateral vascular bundle runs through the entire staminode, but only the phloem elements reach the epidermis in the distal region, where a small protuberance is observed, possibly a reduced anther (Fig. 4i). In all nectar‐producing species, the androecial hood is tightly pressed against the staminal ring and the ovary apex, and pollinators access the nectariferous cavity through a canal originating in the staminal ring.

### Are floral rewards evolutionarily correlated with floral symmetry in Lecythidoideae?

**Fig. 5:**
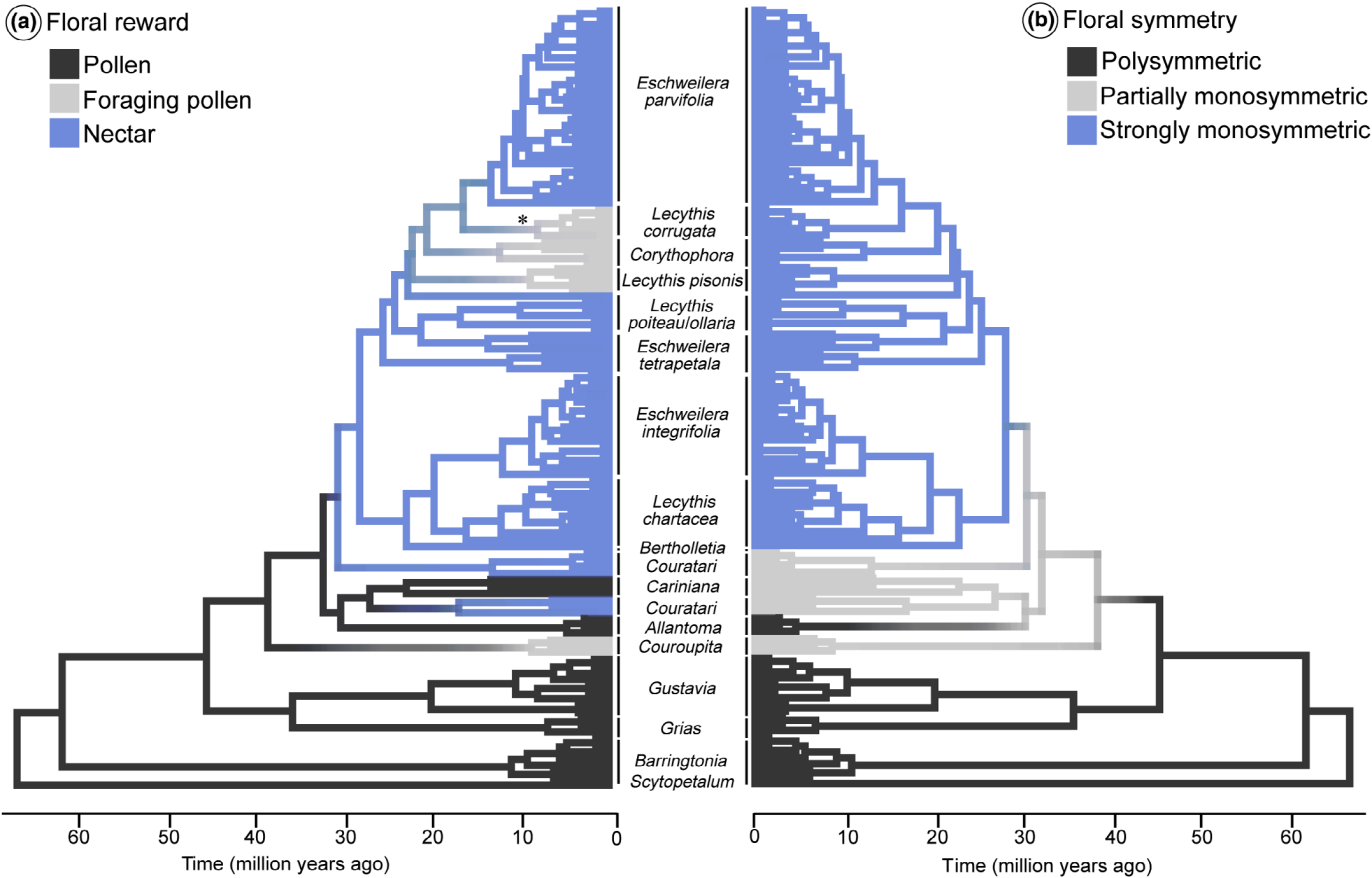
Evolution of floral rewards and floral symmetry in 118 species of Lecythidaceae, showing that monosymmetry evolved once and is correlated with changes in floral rewards from fertile pollen to nectar. (a, b) Ancestral state reconstructions were performed using stochastic character mapping. (a) The posterior density (PD, from 0 to 1) of ancestral lineages having fertile pollen as a reward is indicated in dark gray (i.e. the darker the gray, the greater the probability of rewarding fertile pollen); light gray represents foraging pollen as a reward; blue represents nectar‐rewarding flowers, * indicates the simultaneous occurrence of foraging pollen and nectar in the lineage. (b) The posterior density (PD, from 0 to 1) that ancestral lineages were polysymmetric is indicated in dark gray (i.e. the darker the gray, the more likely they were polysymmetric); light gray represents partially monosymmetric flowers; finally, blue represents strongly monosymmetric flowers. Nectar has evolved at least twice independently in monosymmetric contexts in Lecythidoideae. Taxonomic clades are indicated at the tips of the phylogenetic tree.

By reconstructing the evolutionary history of floral rewards and floral symmetry, our analyses supported the ancestry of polysymmetric flowers with fertile pollen reward in Lecythidoideae (Fig. 5). The kind of reward appears to be a more labile trait than floral symmetry (Table S2). Foraging pollen seems to have evolved at least four times from ancestors that produced fertile pollen and, mainly, nectar (Fig. 5a). Nectar might have evolved twice from fertile pollen‐producing ancestors. Floral symmetry first evolved into partially monosymmetric flowers and later into strongly monosymmetric flowers (Fig. 5b). In the Allantoma clade, there was a reversal from partially monosymmetric flowers to polysymmetric flowers. Interestingly, the stochastic maps indicated a high posterior density that floral monosymmetry arose in an ancestor that had fertile pollen as a reward (Fig. 5). Similarly, nectar had a high probability of evolving in partially monosymmetric flowers.

The results of the analyses of correlated evolution between floral rewards and floral symmetry indicated that the dependent evolution model was preferred over the model in which all changes occur independently of character states. The dependent model yielded a log marginal likelihood of −38.57, whereas the independent model yielded −43.24. The resulting LBF = 9.34 provides very strong support in favor of the dependent model. We found that changes toward rewards, such as foraging pollen and nectar, occur only in the context of monosymmetric flowers (Fig. 5).

### Diversification dynamics

Comparison of the diversification models using AICc and AICw criteria indicated that the MuHiSSE model with hidden states provided the best fit to the data (Table 1). The MuSSE model, which allows for different extinction rates, also received considerable support with similar values. Furthermore, the null BiSSE models, each fitted separately for reward kind and floral symmetry, received greater support than the nonhidden BiSSE models and their HiSSE counterparts (Table 1). These results suggest that the data are best explained by models in which the observed traits do not directly drive diversification but interact with other unmeasured sources of variation.

**Table 1:** Akaike information criterion values (AICc and AICw) for diversification models under the combination of floral rewards and floral symmetry, as well as for each trait separately, in Lecythidoideae, using the diversitree and hisse packages.

Models	AICc	AICw
Character independent	787.93	0.54
MuSSE diff epsilon	788.90	0.33
Null dull	790.85	0.12
BiSSE null reward	775.02	0.54
BiSSE reward	775.66	0.39
HiSSE reward	779.41	0.06
BiSSE null symmetry	751.55	0.69
BiSSE symmetry	753.64	0.24
HiSSE symmetry	756.68	0.05

### Generalized linear mixed models

**Fig. 6:**
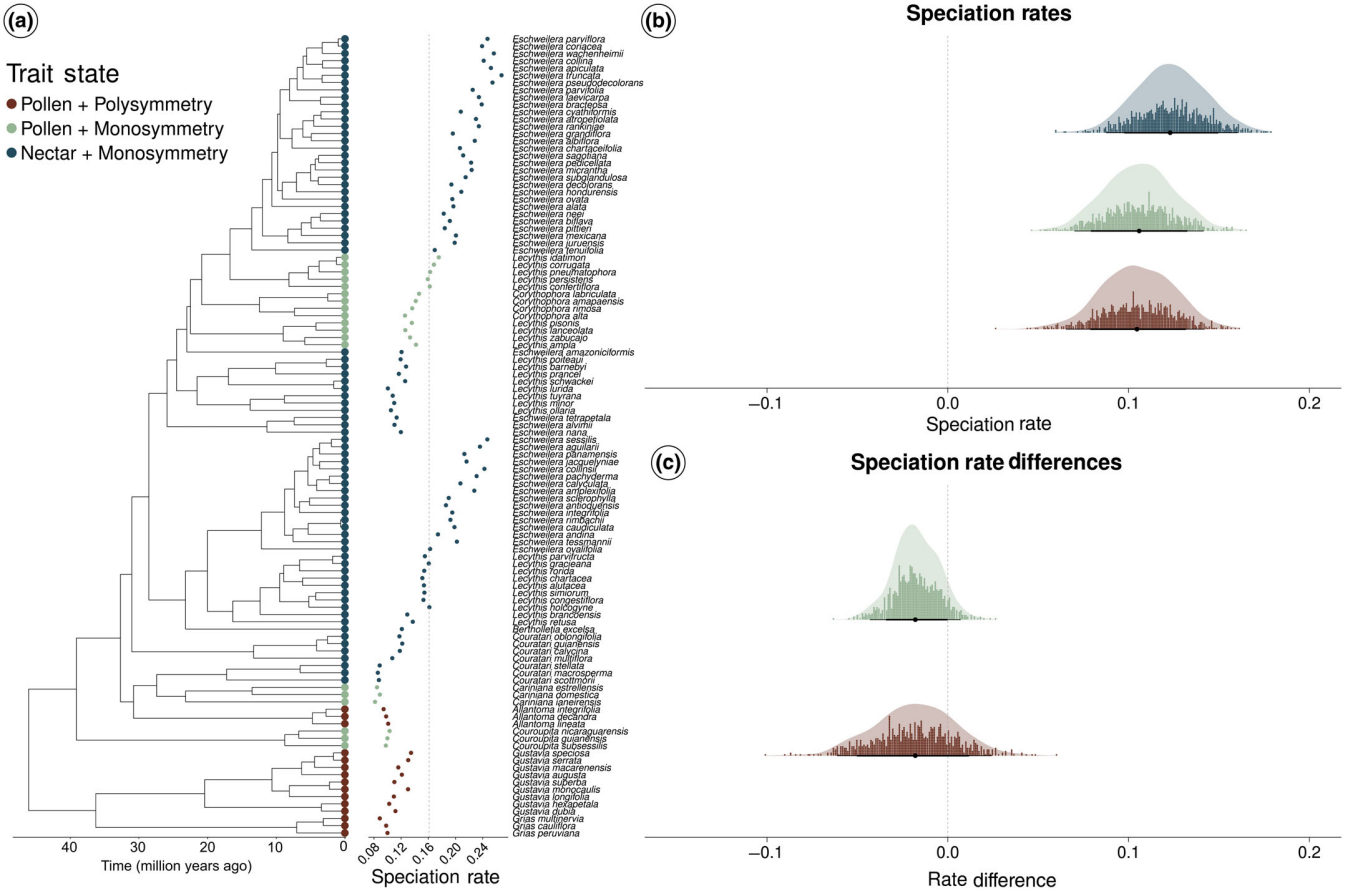
Relationship between combinations of floral traits and speciation rates in Lecythidoideae. (a) Phylogeny of Lecythidoideae displaying the distribution of trait states in the tips and speciation rates. The colors of the symbols at the tips describe the trait state, while both the icon size and the plot to the right show the distribution of speciation rates estimated using CLaDS. (b) Posterior distribution of parameters describing the relationship between speciation rates from CLaDS and trait state. The dots represent the binned parameter values and their respective frequency, whereas the curve denotes the posterior probability density of parameter values. For each state, the dots, thick lines, and thin lines represent, respectively, the median, the 83% and 95% credibility intervals. The only state with a significant effect is ‘Nectar + Monosymmetry’ (top distribution) since it is the only one that neither credibility interval includes 0. (c) Posterior distribution of parameter differences from all states in relation to ‘Nectar + Monosymmetry’. The dots represent the binned parameter values and their respective frequency, whereas the curve denotes the posterior probability density of parameter values. For each state, the dots, thick lines, and thin lines represent, respectively, the median, the 83% and 95% credibility intervals. The only state with a significant effect is ‘Pollen + Polysymmetry’ (bottom distribution) since it is the only one that neither credibility interval includes 0.

The posterior distribution of parameter values indicates that species presenting the syndrome comprising ‘Nectar + Monosymmetry’ have the highest speciation rates (Fig. 6b). The rates for those species are significantly different from the other two, given that there is no overlap between the credibility intervals. For the other two states (namely ‘Pollen + Polysymmetry’ and ‘Pollen + Monosymmetry’), the posterior distribution of parameter values shows a large overlap between each other, indicating that these two states do not differ in terms of speciation rates (Fig. 6c).

## Discussion

Our results show that considering the interactions between traits is essential for understanding the dynamics of species diversification. We found that the evolution of floral rewards is correlated with the evolution of floral symmetry and that these traits have positive effects on the diversification dynamics of Lecythidoideae. Most prominently, nectar production and monosymmetric flowers are associated with increased speciation rates in Lecythidoideae (Fig. 6). Similarly, other factors related to floral rewards and symmetry also appear to influence diversification rates (Table 1). Combinations of traits can be adaptive, and only when these traits are associated can we observe their positive effect on diversification (Anderson *et al*., 2023). Indeed, the concept of the floral syndrome assumes that floral traits are not independent of each other (Vamosi *et al*., 2018; Dellinger, 2020), and the correlation between these traits has already been considered important for diversification dynamics (O'Meara *et al*., 2016). Floral monosymmetry, a low number of stamens, and the presence of a corolla have acted as key innovations in angiosperms, doubling diversification rates in lineages with these three traits together and being more effective than each of them individually (O'Meara *et al*., 2016). In this study, we followed the hypothesis proposed by Anderson *et al*. (2023), which postulates that pollination specialization, driven by the presence of floral monosymmetry, can affect population persistence or speciation rates by increasing reproductive isolation.

### Floral specialization in Lecythidoideae

The structural modification of the androecium in Lecythidoideae allowed the production of different kinds of floral rewards. Structural differences in the stamens and staminodes of this subfamily are consistent with the functional adaptations expected for bee pollination (for review, see Armbruster, 2014; Rosas‐Guerrero *et al*., 2014). In particular, Euglossini bees play an important role in pollinating species with monosymmetric flowers, especially those that produce nectar (Prance, 1976; Mori & Boeke, 1987; Mori & Prance, 1987). There is evidence of coevolution between these flowers and Euglossini bees, as both groups are restricted to the Neotropics and show considerable overlap in their current distributions (Mori & Boeke, 1987). Within the subfamily Lecythidoideae, the androecial hood found in monosymmetric species serves two fundamental functions: It acts as a signal for the floral structure and as a landing platform for the bees, directing them inside of the flower (Prance, 1976; Tsou & Mori, 2007). The hood allows access only to pollinators strong enough to push it, ensuring that the bees keep their dorsal regions pressed against the fertile anthers of the staminal ring (Prance, 1976; Mori & Prance, 1990). Furthermore, to pollinate efficiently, bees must have tongues long enough to reach the nectar located at the end of the hood spiral (Mori & Boeke, 1987).

Our structural findings provide an important perspective into the evolution of nectar production from ancestral pollen‐producing species in Lecythidoideae. These species exhibit a diversity of reward strategies, ranging from flowers that offer only fertile pollen without intrafloral differentiation to those that offer both fertile and foraging pollen within the same flower (Mori & Boeke, 1987). In these species, the stamens and the fertile and infertile pollen are morphologically and physiologically differentiated (as in *Couroupita guianensis*, *Corythophora alta*, and *Lecythis pisonis*) (Mori & Orchard, 1979; Mori *et al*., 1980). Some species appear to represent an intermediate evolutionary stage, offering both pollen and nectar, suggesting an evolutionary transition from pollen‐based to nectar‐based rewards (Mori & Boeke, 1987). Although some species, such as those belonging to the Lecythis corrugata clade, may offer both foraging pollen and nectar as rewards to pollinators, evidence from reproductive biology studies suggests that the efficient pollinators of these species enter flowers primarily to collect foraging pollen (Mori *et al*., 2010). It is therefore likely that this trait is under intense selective pressure. The presence of both rewards in species such as *Lecythis poiteaui* seems to reflect a remnant stage of this transition, as this species is pollinated by nectar‐seeking bats (Mori & Boeke, 1987; Prance & Mori, 1998; Mori *et al*., 2010). In this species, the staminodes that secrete nectar at the base do not produce differentiated pollen in their anthers (Fig. S2i). Although the floral morphology of Lecythidoideae species has been studied and elucidated due to their highly specialized floral structures, our results are novel in providing anatomical evidence of the stamens and the unusual nectaries. We demonstrate that the evolution of nectar‐producing species is associated with the reduction in the number of fertile stamens due to the sterilization of the stamens in the androecial hood, and eventually the complete transformation of these stamens into nectaries.

In addition to the coevolution of the androecial hood in Lecythidoideae, other factors related to structural changes in the androecium may be associated with the specialized pollination pattern in monosymmetric species. For instance, in *Bertholletia* and some species of *Lecythis*, nectar secretion occurs at the base of the staminodes within the androecial hood (Figs 3d, S2), making access difficult and promoting specificity in pollen transfer (Prance, 1976; Mori *et al*., 1978; Mori & Boeke, 1987; Mori & Prance, 1990). The nectar composition of *B. excelsa* has been shown to be predominantly sucrose, an unusual characteristic among angiosperms (Baker & Baker, 1983; Galetto & Bernardello, 2003). This finding supports our hypothesis about the dynamics of nectar secretion in species with peripheral vascular bundles (Figs 3, S2), since sucrose is the main sugar transported by the phloem conducting elements (Giaquinta, 1977, 1983; Liu *et al*., 2012; Zhang & Turgeon, 2018; Braun, 2022). The secretion of sucrose‐rich nectar in *B. excelsa* suggests that this sugar is released directly from the sieve tube elements into the external medium or into the cytoplasm of the secretory cells surrounding the vascular bundle. The presence of sugars outside of the vascular bundle can decrease the water potential, causing water to move from the tracheal elements to the external environment, resulting in nectar formation (Fig. 3h). This process is facilitated by the absence of a cuticular barrier in this region (Fig. 3g). To our knowledge, this unusual mechanism of nectar secretion has not been described before. Furthermore, Euglossini bees tend to prefer flowers that produce sucrose‐rich nectar (Roubik *et al*., 1995), which explains the specific relationship between these flowers and their pollinators. For a better understanding of the relationship between nectar and its pollinators, and considering the new structural perspective of the nectaries in Lecythidoideae, detailed and comparative studies of the nectar secretion process are needed. These should include the identification of the cellular organelles involved in nectar synthesis and release, as well as analyses of the sugar composition and the amount of nectar produced. Overall, the evolution of rewards for foraging pollen and nectar has led to more specialized pollination, accompanied by an increase in the structural complexity of the androecium. The evolution of a more closed androecium, which restricts access to nectar, ensures that only effective pollinators, such as Euglossini bees, can access it. This, combined with floral monosymmetry, has restricted access to the rewards offered.

### How did correlation affect diversification dynamics?

Using evolutionary correlation analysis, we identified a link between the evolution of floral reward and the evolution of symmetry. Previous studies on polysymmetric genera of Lecythidoideae showed that radial symmetry does not predictably guide pollinators to the flower (Tsou & Mori, 2007). By contrast, monosymmetric flowers predictably orient pollinators (Tsou & Mori, 2007). Flower monosymmetry plays a critical role in guiding Euglossini bees to nectar and foraging pollen, as bilaterally symmetrical patterns indicate the location of floral rewards (Giurfa *et al*., 1996, 1999). Our results show that in Lecythidoideae, monosymmetry affecting the perianth and androecium evolved before rewards such as foraging pollen and nectar, allowing the subsequent evolution and persistence of these rewards in a context where monosymmetry affects all floral organ sets (Figs 5, S3). We propose that the initial evolution toward a monosymmetric state in Lecythidoideae flowers promoted the exploration of new traits, such as the reduction in the number of fertile stamens and the change in the kind of floral rewards. Apparently, the expression of abaxial dominance in flower development and the consequent emergence and refinement of an androecial hood facilitated structural changes in the stamens, rendering them infertile and/or nectaries (Tsou & Mori, 2007). Therefore, the evolution of the flower to a monosymmetric state has been fundamental for understanding which individual traits tend to emerge first and allow the evolution of other traits, thereby altering diversification rates. This is currently one of the key questions in macroevolutionary studies of angiosperms (Sauquet & Magallón, 2018).

Floral specialization, driven by the evolution of morphological structures adapted for pollination, has long been associated with increased speciation rates in angiosperms (Grant, 1994; Rosas‐Guerrero *et al*., 2011; Arceo‐Gómez *et al*., 2016; Ling *et al*., 2025). These adaptations often lead to highly specific interactions with pollinators, thus promoting pre‐zygotic reproductive isolation among sympatric or closely related species (Armbruster, 2014). Indeed, several studies have emphasized the role of floral specialization in shaping diversification dynamics. For example, lineages with nectar spurs in Antirrhineae (Plantaginaceae) exhibited higher speciation rates, which initially supported the key innovation hypothesis for this trait (Fernández‐Mazuecos *et al*., 2019). However, using a hidden state approach, the same study found that nectar spurs alone were only marginally associated with increased diversification. Our fit analyses of the MuHiSSE models reveal a similar pattern, where lineage diversification can be explained by a complex interplay of unmeasured biotic and abiotic factors. Similar hypotheses about diversification have been proposed for anther glands in Calophyllaceae, which produce oils and resins as rewards and establish specific relationships with pollinators (Cabral *et al*., 2021). These glands played a significant role in promoting positive changes in the net diversification rates of the family (Cabral *et al*., 2021). Similarly, glandular trichomes that secrete oils, a key trait for adaptation to specific pollination systems in American Iridoideae, appear to have contributed significantly to increased speciation rates in this group (Chauveau *et al*., 2012). In general, plants and pollinators interact through traits that influence the frequency and effectiveness of pollination (Sletvold, 2019). These interactions depend mainly on the rewards that flowers provide to pollinators, such as nectar and pollen (Parachnowitsch *et al*., 2019).

Null BiSSE models, in which there is no variation associated with the trait states, showed slightly stronger support than trait‐dependent diversification models when reward type and floral symmetry were analyzed separately, suggesting that each trait alone is insufficient to explain variation in diversification rates (Table 1). However, when the interaction between these traits was considered within the MuSSE framework, we found that combinations of floral traits may play a role in diversification dynamics (Table 1). In the MuSSE framework, models incorporating the interaction between either floral traits or hidden states outperformed the null model, indicating that heterogeneity in diversification rates is driven either by the combination of floral traits or by unmeasured variables, potentially reflecting complex interactions between biotic and abiotic factors (such as Lagomarsino *et al*., 2016; Dellinger *et al*., 2024). This interpretation is supported by the mcmcglmm results, which revealed large uncertainties in the posterior distribution of diversification rates, suggesting the influence of additional factors not explicitly considered in the models. Furthermore, trait‐independent methods such as BAMM (Vargas & Dick, 2020) and CLaDS (Fig. 6a) also detected pronounced variation in speciation rates across the Lecythidoideae phylogeny. In particular, lineages with nectar production and monosymmetric flowers consistently exhibited higher speciation rates than those with other forms of symmetry or kinds of floral rewards (Fig. 6a). This pattern is consistent with the key synnovation hypothesis, which suggests that this combination of traits can create new ecological opportunities and promote diversification (Donoghue & Sanderson, 2015).

The results of the trait‐dependent diversification analyses indicated that variation in diversification rates cannot be explained by the mapped floral traits alone. However, the specific support for the MuSSE model with different extinction rates reinforces the existence of heterogeneity in extinction rates across character states, suggesting that floral traits such as production of nectar and monosymmetry indirectly influence lineage persistence. This heterogeneity may reflect different degrees of floral specialization, which in turn affect species' vulnerability to local extinctions or environmental changes (Armbruster & Muchhala, 2009; Armbruster, 2016). The interaction between nectar production and monosymmetry appears to enhance pollen transfer efficiency, potentially leading to greater reproductive success and consequently increased lineage persistence over time. In this case, persistence may play a more important role in generating diversification than speciation itself (Anderson *et al*., 2023). Still, the positive effect of floral monosymmetry on the diversification of a clade depends on the environmental context (Donoghue, 2005; Vamosi & Vamosi, 2011). In more stable environments, such as tropical rainforests, the center of diversity for Lecythidoideae (Mori *et al*., 2017), pollination failures are less frequent, strengthening the effect of floral symmetry on reproductive isolation and, therefore, speciation (Anderson *et al*., 2023). This pattern is similar to that observed in other groups, such as birds, where specialist species tend to have higher fitness than generalists in stable environments because they are better adapted to exploit specific resources (Burin *et al*., 2016). However, despite these findings, accurately inferring the mechanisms underlying the observed patterns of diversification remains challenging, mainly due to limitations in the ability of current models to estimate extinction rates. For example, the two models with similar support to the data produce markedly different extinction estimates, ranging from 0 to 0.8 for the mapped states, but virtually zero in the hidden‐state model. This contrast reflects an ongoing debate regarding the limitations of molecular phylogenies in estimating extinction rates (Rabosky, 2010, 2016; Beaulieu & O'Meara, 2015), emphasizing the need for cautious interpretation of the results.

Lastly, it is important to acknowledge the natural limitation related to the size of the analyzed clade (for a review, see Helmstetter *et al*., 2023), and that we are well aware of our limited phylogenetic sampling. This limitation might either favor the inference of false associations (when the sampling is phylogenetically clustered) or hinder our ability to detect significant associations (when there is too little information for good parameter estimation). These issues are certainly alleviated in our case, given that our sampling does not show strong phylogenetic biases and that we do find associations between trait states and rates. Therefore, our results should be interpreted with this caveat in mind. Nevertheless, it is important to stress that the multiple analytical routes taken here do not contradict each other, but rather indicate different levels of association between the floral traits and the diversification rates. Evolutionary datasets are often affected by pseudoreplication problems (Maddison & FitzJohn, 2015; Rabosky & Goldberg, 2015), and to address these potential concerns, we used hidden‐states models to assess the influence of mapped and unmeasured traits in our analyses. As the hidden‐states models indicated that diversification in Lecythidoideae is shaped not only by our trait of interest but also by interactions with unobserved factors, likely including patterns of pollination specialization and occurrence in stable tropical forest environments, we believe our results contribute to the broader debate on the mechanisms underlying diversification dynamics in angiosperm lineages. Despite this, Anderson *et al*. (2023) argue that smaller scale individual studies provide greater analytical control and a better understanding to interpret results, allowing the exploration and comparison of factors influencing diversification across groups and environmental conditions. To confirm whether the patterns we observed are consistent in other lineages, we recommend further studies that examine the correlation between traits, especially the interaction between floral traits, to address broader questions about diversification.

### Conclusions

Our study provides an interesting framework for investigating evolutionarily correlated traits, which is essential for understanding the diversification dynamics of angiosperm lineages (Donoghue & Sanderson, 2015; Sauquet & Magallón, 2018; Zenil‐Ferguson *et al*., 2019; Anderson *et al*., 2023). In Neotropical Lecythidaceae, floral rewards are produced by structures derived from modifications of the androecium, resulting in the gradual sterilization of male function. The evolution of floral rewards correlates with the evolution of floral symmetry, with nectar production being most closely associated with highly monosymmetric floral forms. Our results suggest that other factors associated with floral rewards and symmetry, although not considered in this study, may have influenced diversification rates. The interplay between specialized floral traits, biotic and abiotic factors, and diversification rates in Lecythidoideae contributes to a deeper understanding of the evolutionary processes that shaped angiosperm diversity. Additional studies are needed to assess which traits may evolve in a correlated manner and influence diversification rates in plant lineages. Understanding how these traits interact and influence diversification can help elucidate the evolution and maintenance of angiosperm diversity, as well as the evolutionary processes that have shaped the diversity of forms and reproductive strategies observed in these plants.

## Competing interests

None declared.

## Author contributions

DG, GB, SMC‐G and EDT contributed to the conception of the research. DG collected the data. DG and GB conducted all data analyses. All authors contributed to the interpretation of the results. DG and GB prepared the figures and wrote the manuscript. All authors contributed to revisions and gave final approval for publication.

## Disclaimer

The New Phytologist Foundation remains neutral with regard to jurisdictional claims in maps and in any institutional affiliations.

## Supporting information


**Dataset S1** Original images obtained by SEM.

**Dataset S2** List of taxa for reversible‐jump MCMC framework to correlated evolution.

**Fig. S1** Androecium of Lecythidoideae.
**Fig. S2** Structural characterization of nectaries in species of the genus *Bertholletia* and *Lecythis* using light microscopy.
**Fig. S3** Simmap of ancestral state estimations for kinds of floral reward and floral symmetry.
**Table S1** Species vouchers.
**Table S2** Estimated number of transitions between floral rewards and floral symmetry in Lecythidoideae.
Please note: Wiley is not responsible for the content or functionality of any Supporting Information supplied by the authors. Any queries (other than missing material) should be directed to the *New Phytologist* Central Office.

## Data Availability

The data that support the findings of this study are openly available in GitHub at https://github.com/gburin/pollination‐syndromes‐lecythidoideae.git.
