# Supplementary figures and images for: Interaction between floral rewards and floral symmetry shapes diversification dynamics in Amazonian trees
Source: New Phytol. 2025 Oct 8;248(6):3297–311. doi: 10.1111/nph.70623 (PMC12630423; doi:10.1111/nph.70623)

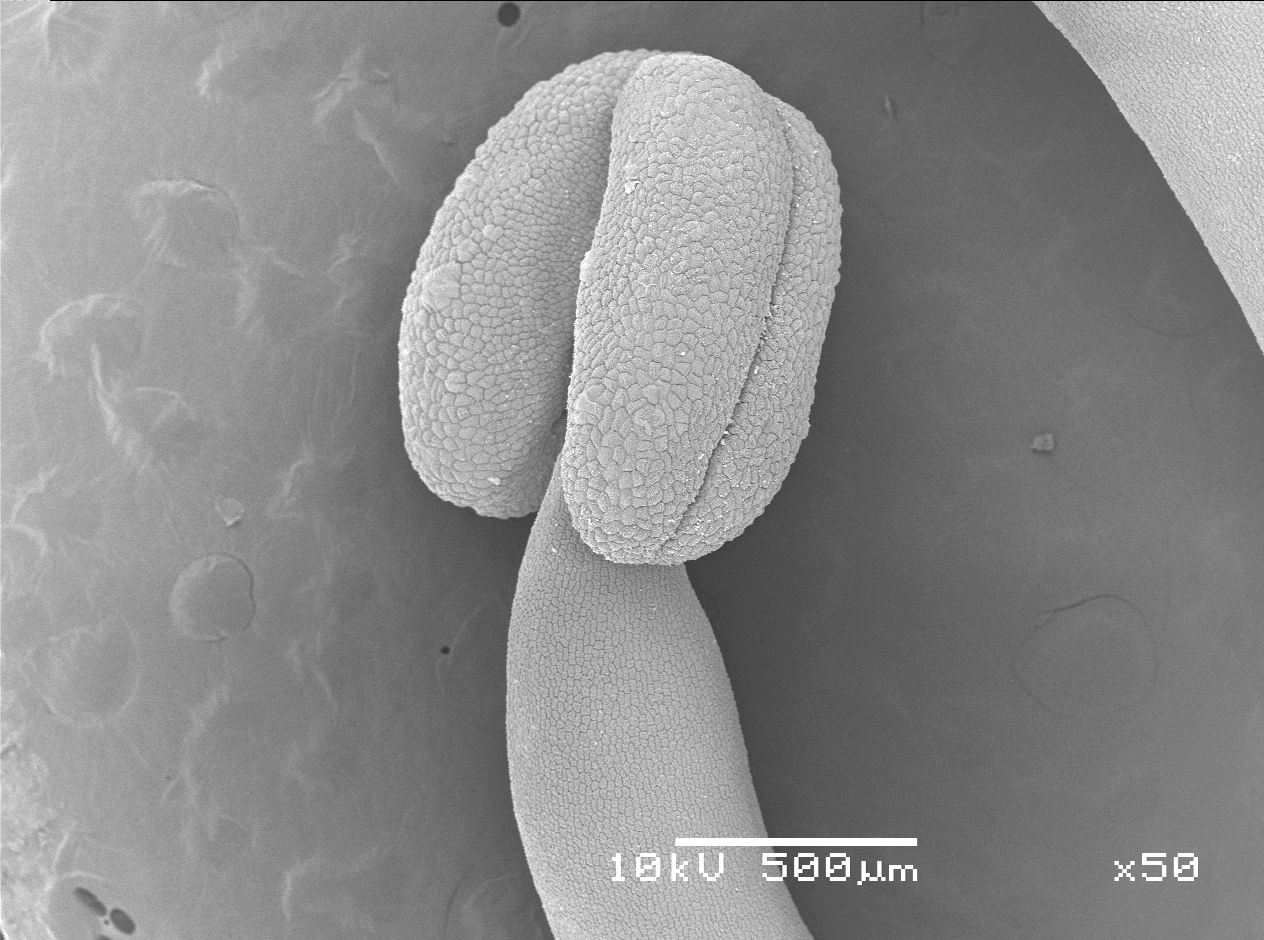

Supplement: Supplementary file 1 — Dataset S1 Original images obtained by SEM. [file NPH-248-3297-s001.zip › SEM Images/Image 2a.TIF]

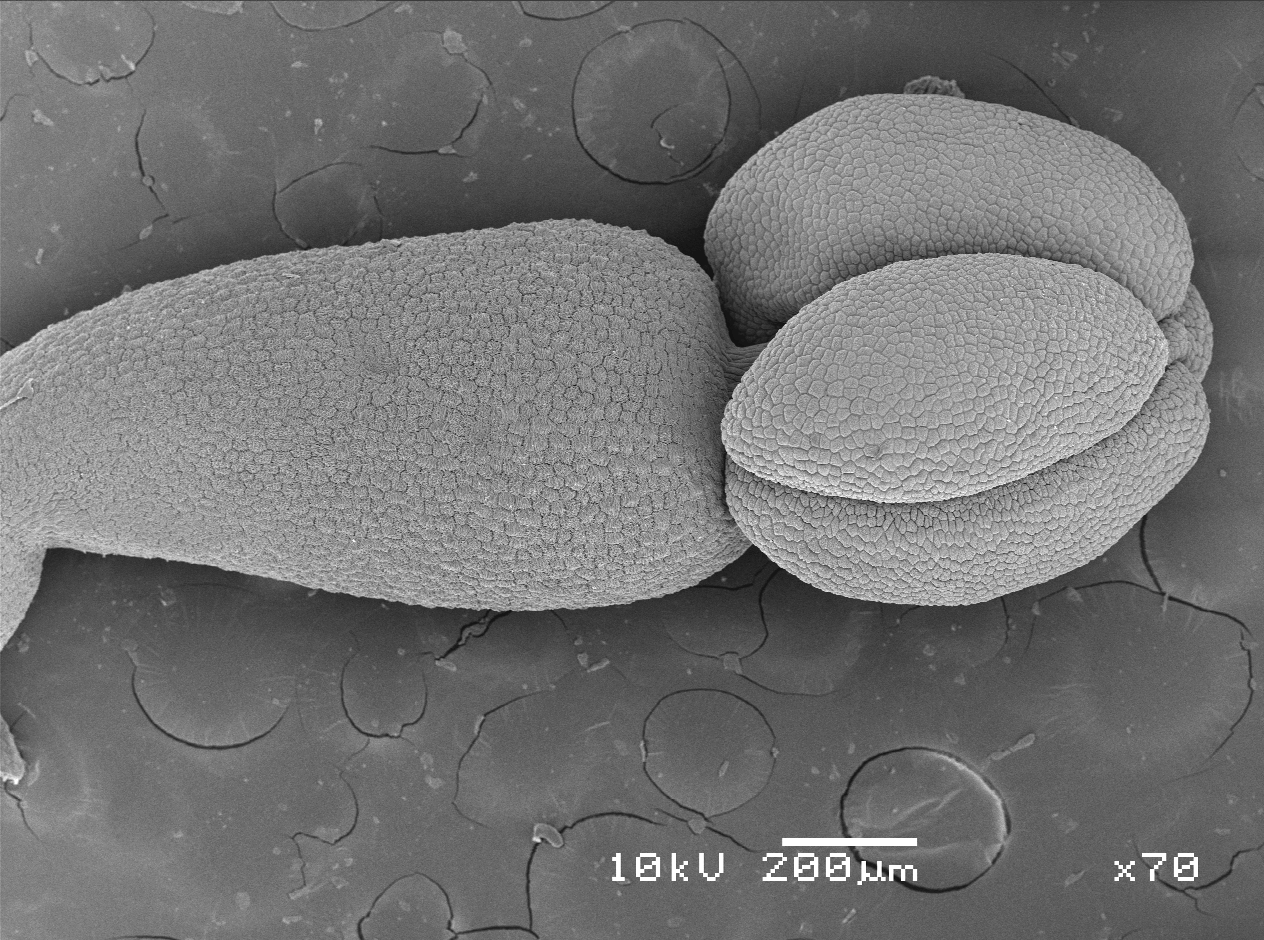

Supplement: Supplementary file 1 — Dataset S1 Original images obtained by SEM. [file NPH-248-3297-s001.zip › SEM Images/Image 2c_C. guianensis.TIF]

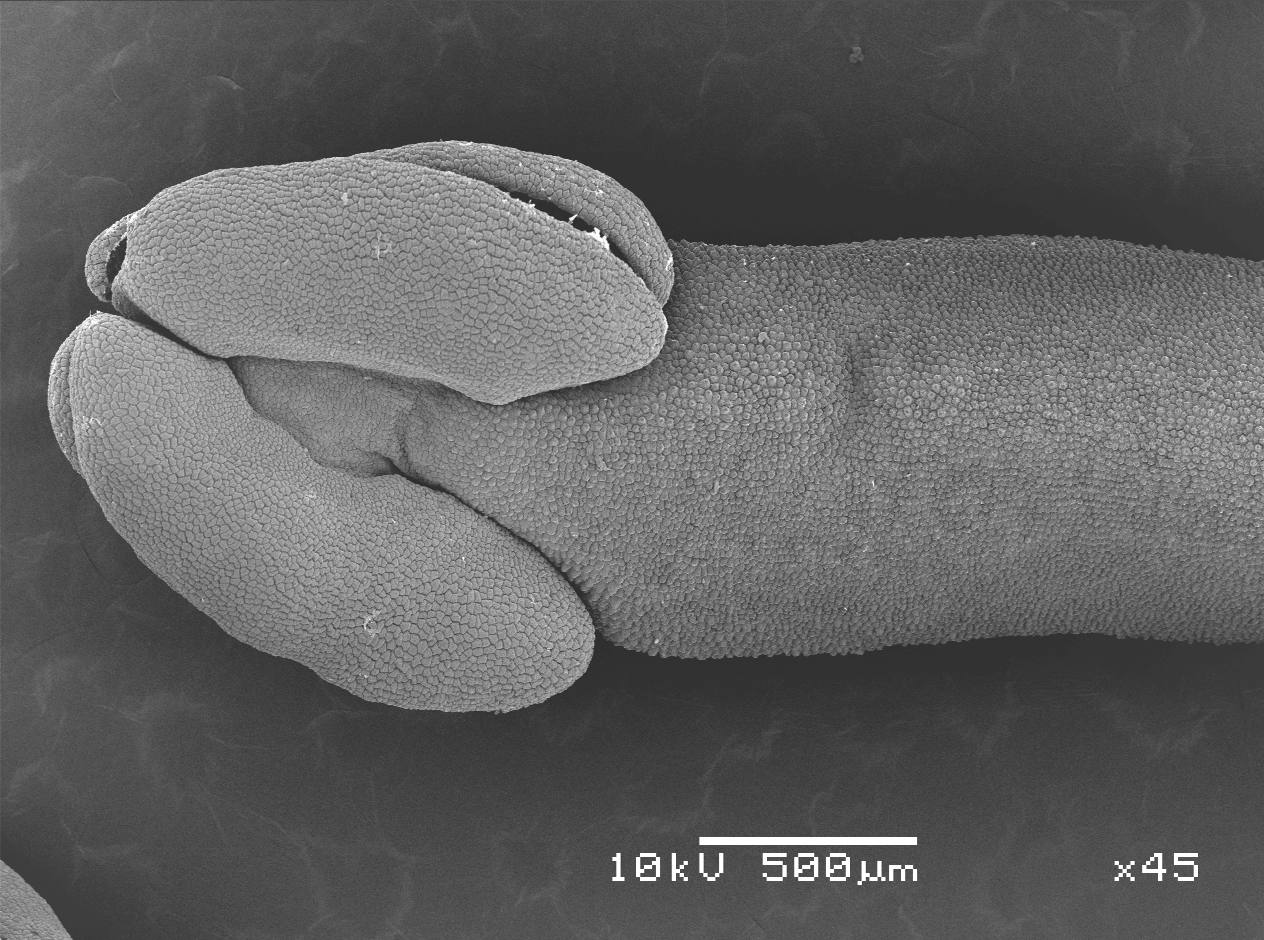

Supplement: Supplementary file 1 — Dataset S1 Original images obtained by SEM. [file NPH-248-3297-s001.zip › SEM Images/Image 2e_C. guianensis.TIF]

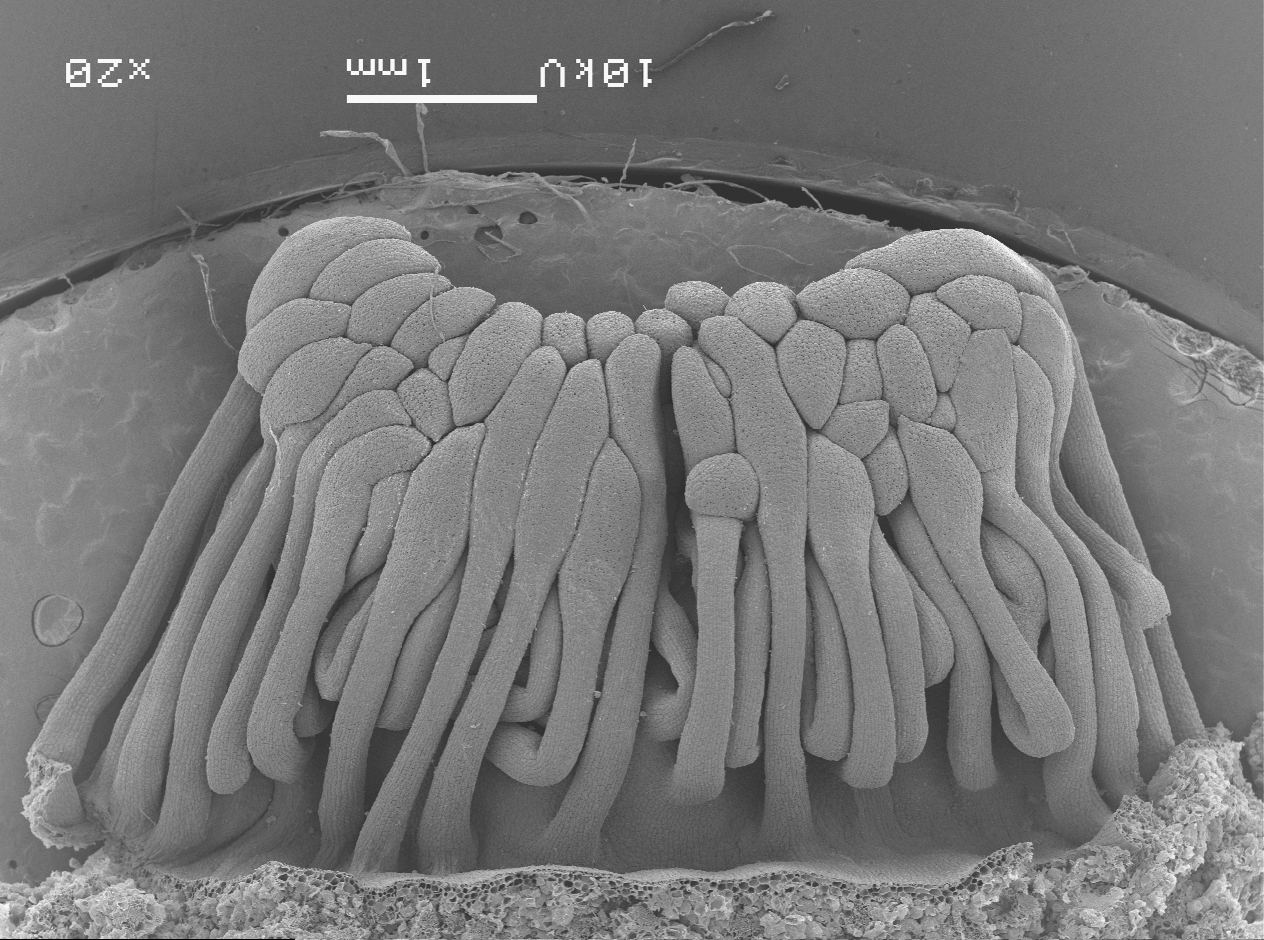

Supplement: Supplementary file 1 — Dataset S1 Original images obtained by SEM. [file NPH-248-3297-s001.zip › SEM Images/Image 3a_C. asterotricha.TIF]

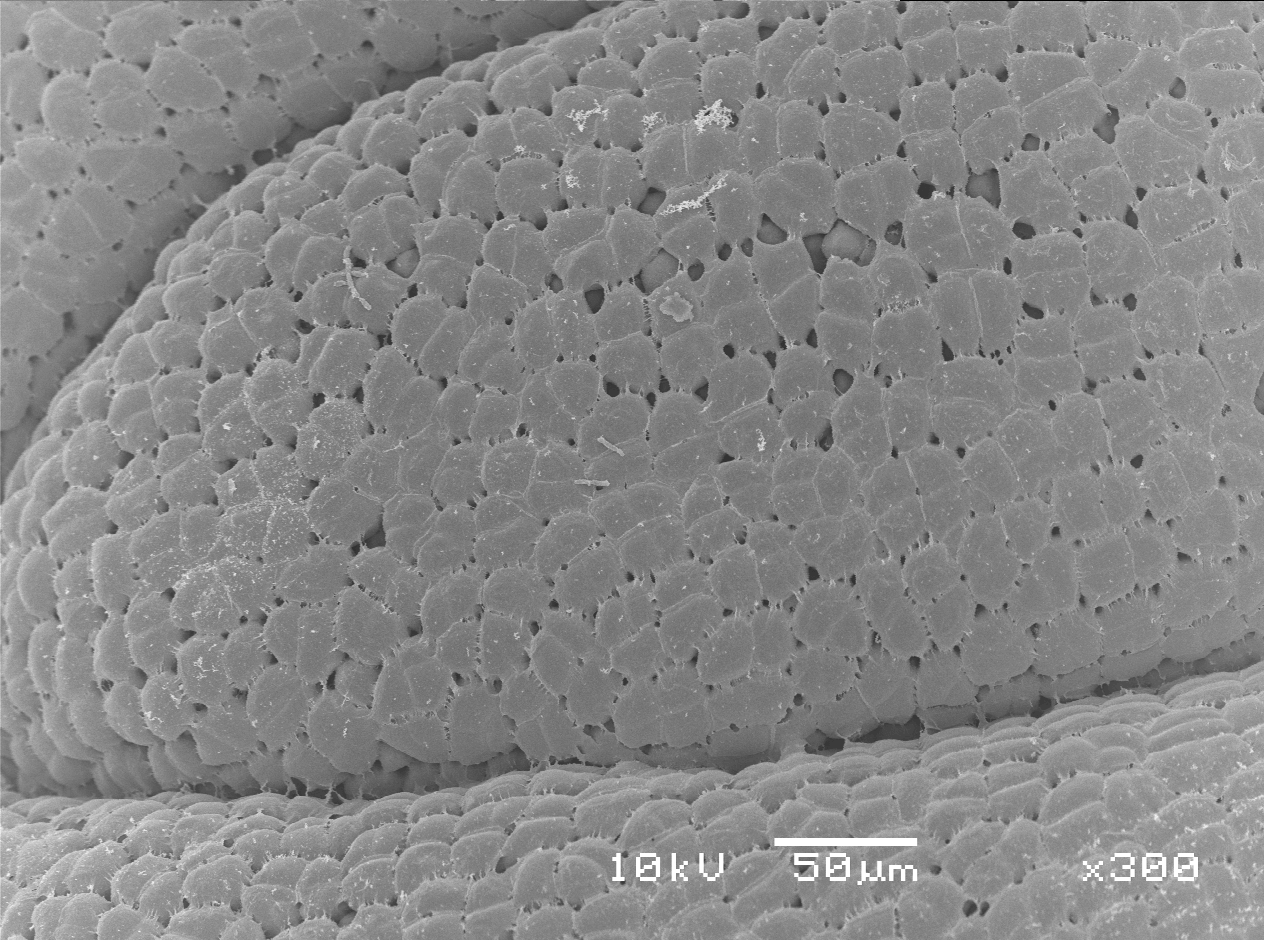

Supplement: Supplementary file 1 — Dataset S1 Original images obtained by SEM. [file NPH-248-3297-s001.zip › SEM Images/Image 3c_C. asterotricha.TIF]

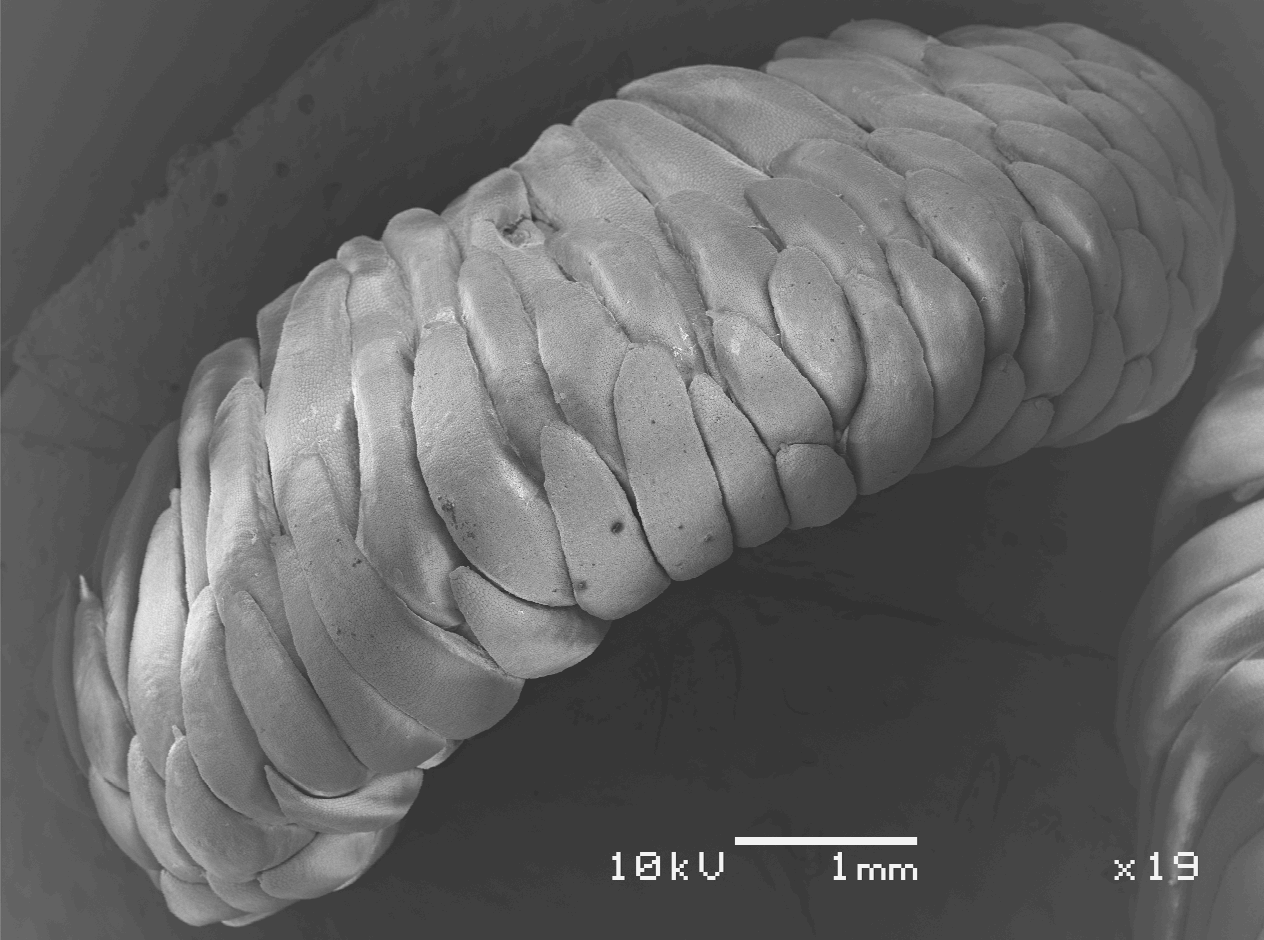

Supplement: Supplementary file 1 — Dataset S1 Original images obtained by SEM. [file NPH-248-3297-s001.zip › SEM Images/Image 4d_E. atropetiolata.TIF]

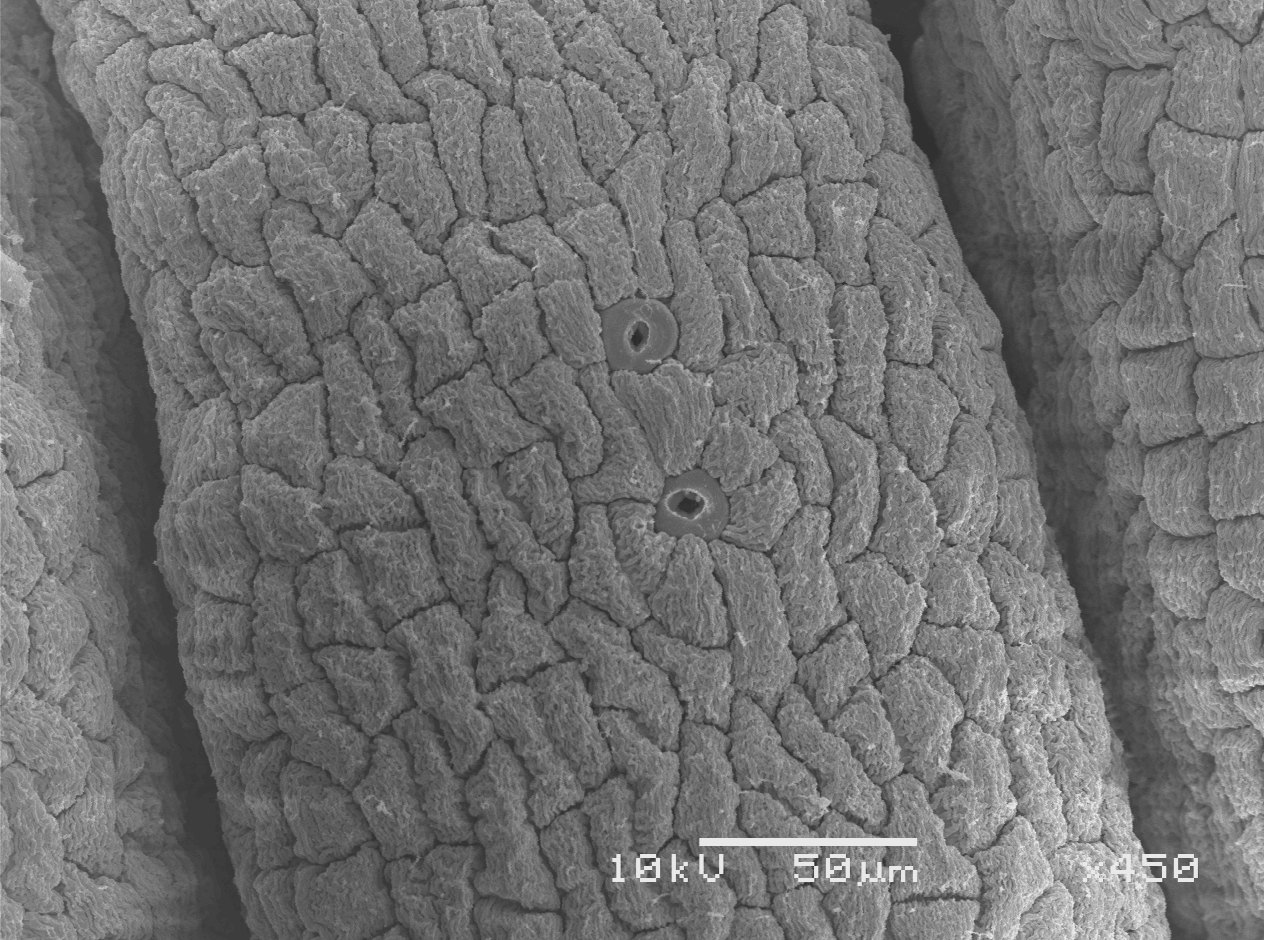

Supplement: Supplementary file 1 — Dataset S1 Original images obtained by SEM. [file NPH-248-3297-s001.zip › SEM Images/Image 4h_E. atropetiolata.TIF]

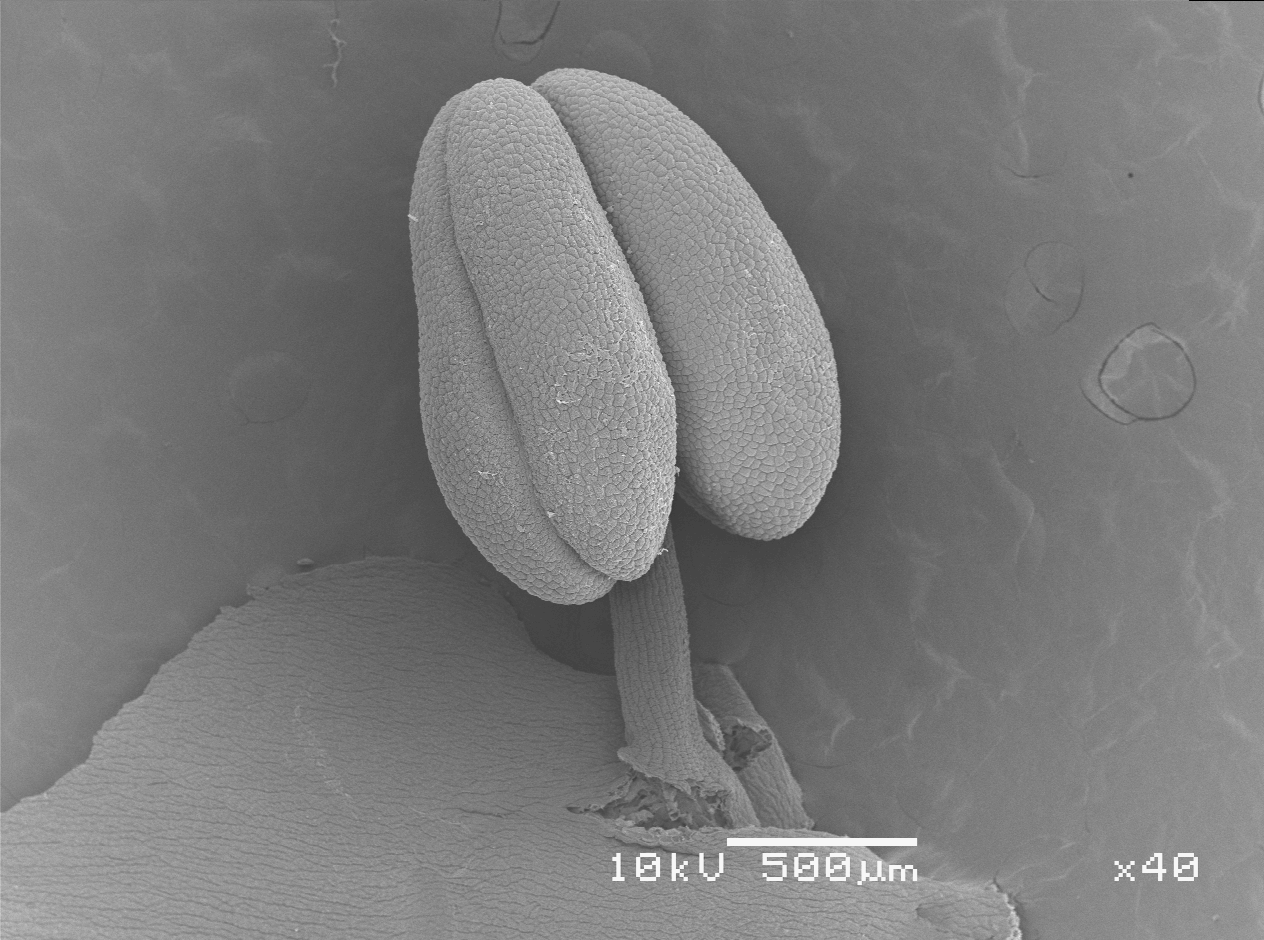

Supplement: Supplementary file 1 — Dataset S1 Original images obtained by SEM. [file NPH-248-3297-s001.zip › SEM Images/Image S1a_C. asterotricha.TIF]

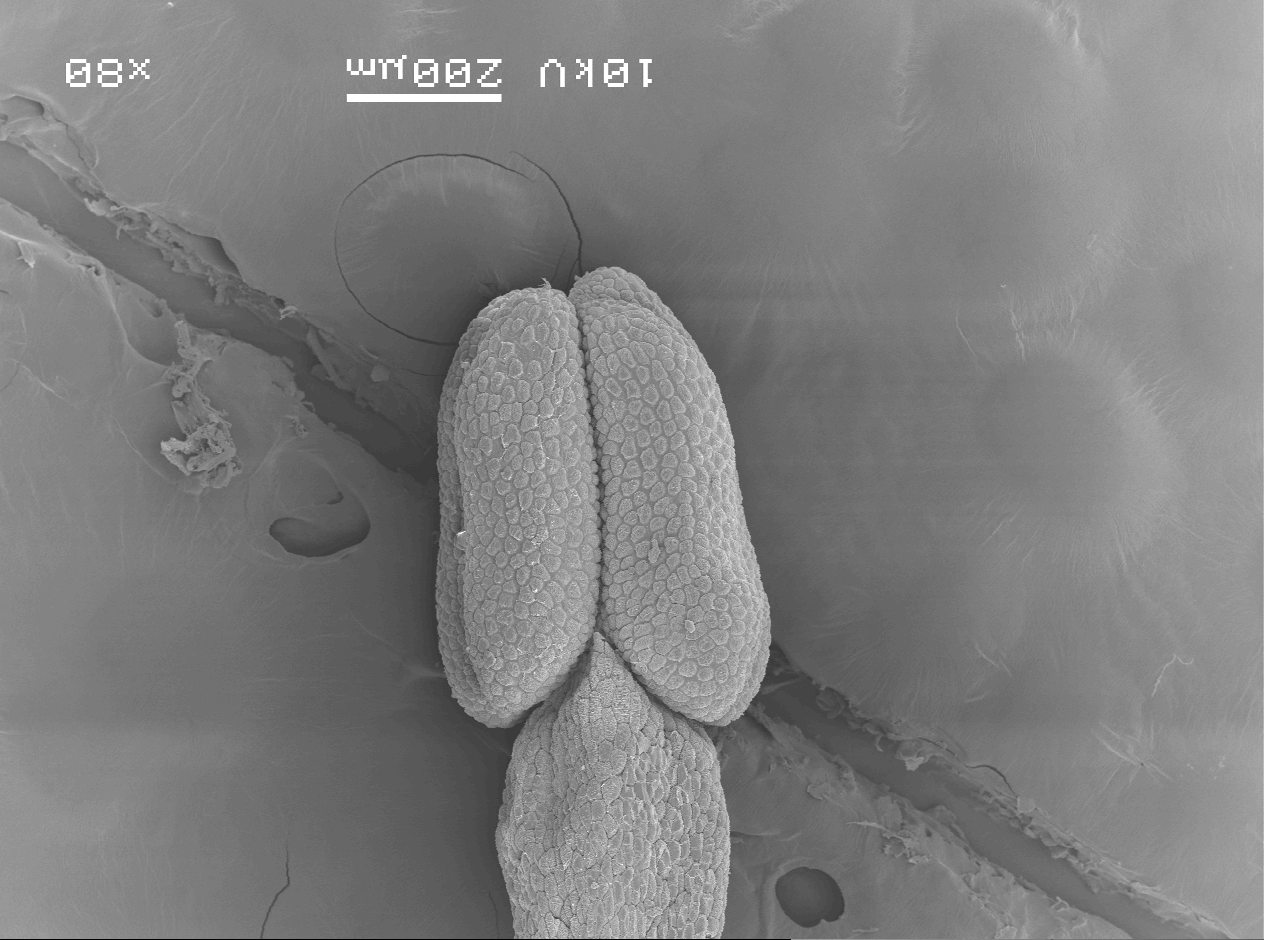

Supplement: Supplementary file 1 — Dataset S1 Original images obtained by SEM. [file NPH-248-3297-s001.zip › SEM Images/Image S1c_C. alta.TIF]

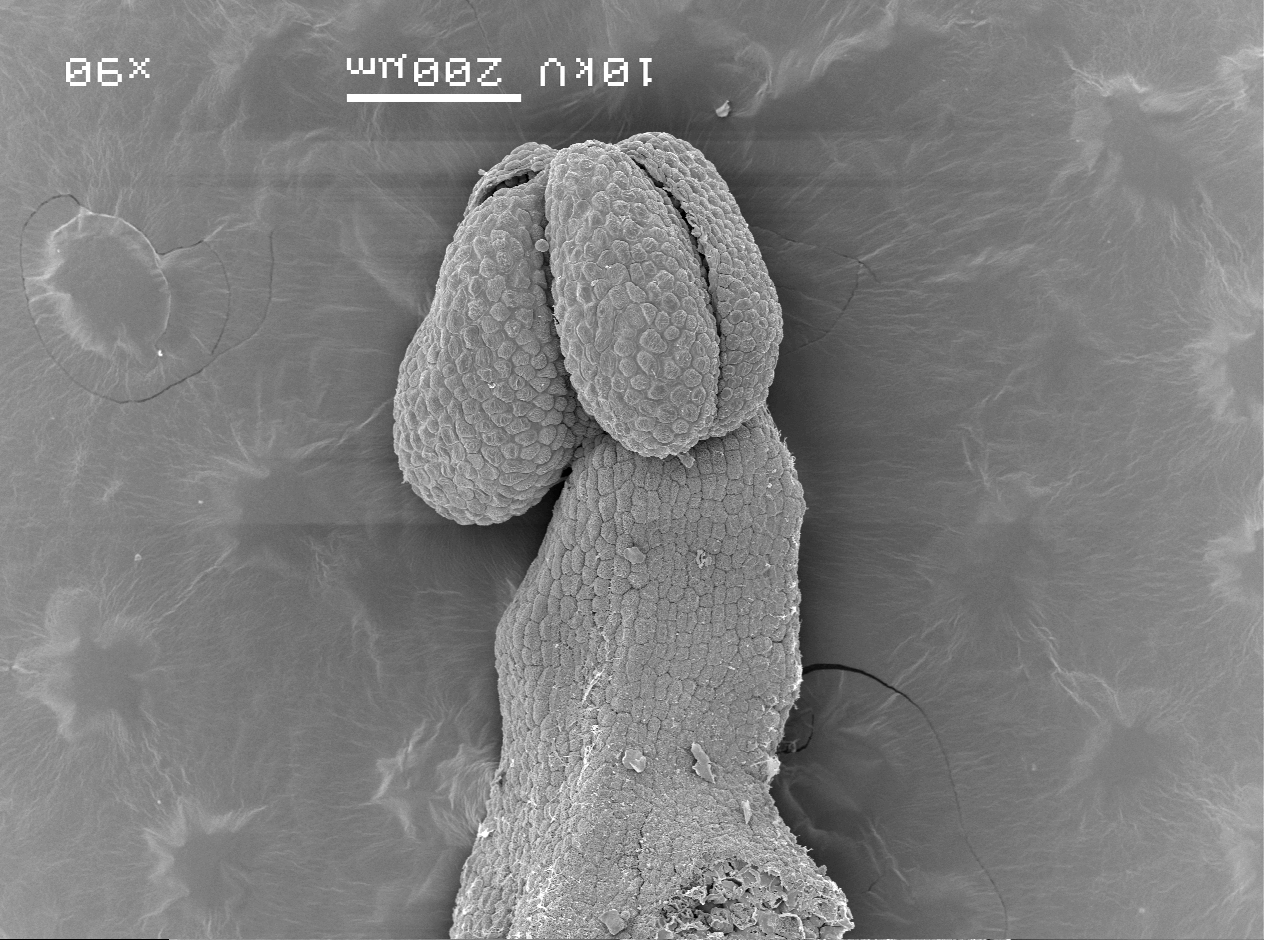

Supplement: Supplementary file 1 — Dataset S1 Original images obtained by SEM. [file NPH-248-3297-s001.zip › SEM Images/Image S1e_C. alta.TIF]
