# Supplementary material for: Interaction between floral rewards and floral symmetry shapes diversification dynamics in Amazonian trees
Source: New Phytol. 2025 Oct 8;248(6):3297–311. doi: 10.1111/nph.70623 (PMC12630423; doi:10.1111/nph.70623)
Supplement: Supplementary file 3 — Fig. S1 Androecium of Lecythidoideae. Fig. S2 Structural characterization of nectaries in species of the genus Bertholletia and Lecythis using light microscopy. Fig. S3 Simmap of ancestral state estimations for kinds of floral reward and floral symmetry. Table S1 Species vouchers. Table S2 Estimated number of transitions between floral rewards and floral symmetry in Lecythidoideae. Please note: Wiley is not responsible for the content or functionality of any Supporting Information supplied by the authors. Any queries (other than missing material) should be directed to the New Phytologist Central Office. [file NPH-248-3297-s003.pdf]

New Phytologist

New Phytologist Supporting Information

Article title: **Interaction between floral rewards and floral symmetry shapes diversification dynamics in Amazonian trees**

Authors: Diego Graciano, Gustavo Burin, Sandra Maria Carmello-Guerreiro and Elisabeth Dantas Tölke

Article acceptance date: 15 September 2025

The following Supporting Information is available for this article:

Table S1 Species vouchers.
Fig. S1 Androecium of Lecythidoideae.
Fig. S2 Structural characterization of nectaries in species of the genus *Bertholletia* and *Lecythis* using light microscopy.
Fig. S3 Simmap of ancestral state estimations for kind of floral reward and floral symmetry.
Table S2 Estimated number of transitions between floral rewards and floral symmetry in Lecythidoideae.
Dataset S1 Original images obtained by SEM.
Dataset S2 List of taxa for reversible-jump MCMC framework to correlated evolution.

Table S1 Species collected for this study with the respective location and voucher.

**Species**	**Location**	**Voucher**
Bertholletia excelsa Bonpl.	Brazil, Manaus – Amazonas, INPA	INPA 249224
Corythophora alta R. Knuth	Brazil, Manaus – Amazonas, Reserva Florestal Adolfo Ducke	INPA 216695
Couratari asterotricha Prance	Brazil, Campinas – São Paulo, Instituto Agronômico de Campinas, Fazenda Santa Elisa	UEC 208785
Couroupita guianensis Aubl.	Brazil, Campinas – São Paulo, UNICAMP	UEC 208782
Eschweilera atropetiolata S. A. Mori	Brazil, Manaus – Amazonas, Reserva Florestal Adolfo Ducke	INPA 216650
Eschweilera collina Eyma	Brazil, Manaus – Amazonas, Reserva Florestal Adolfo Ducke	INPA 215319
Eschweilera coriacea (DC.) S. A. Mori	Brazil, Manaus – Amazonas, Reserva Florestal Adolfo Ducke	INPA 290583
Eschweilera grandiflora (Aubl.) Sandwith	Brazil, Manaus – Amazonas, Reserva Florestal Adolfo Ducke	INPA 216653
Eschweilera pseudodecolorans S. A. Mori	Brazil, Manaus – Amazonas, Reserva Florestal Adolfo Ducke	INPA 215317
Eschweilera truncata A. C. Sm.	Brazil, Amazonas, Projeto Dinâmica Biológica dos Fragmentos Florestais	INPA 224504
Eschweilera wachenheimii (Benoist) Sandwith	Brazil, Manaus – Amazonas, Reserva Florestal Adolfo Ducke	INPA 215300
Grias neuberthii J. F. Macbr.	Brazil, Rio de Janeiro, Jardim Botânico do Rio de Janeiro	HPL 3476
Lecythis gracieana S. A. Mori	Brazil, Amazonas, Projeto Dinâmica Biológica dos Fragmentos Florestais	NY 810312
Lecythis poiteaui O. Berg	Brazil, Manaus – Amazonas, Reserva Florestal Adolfo Ducke	IAN 185372
Lecythis prancei S. A. Mori	Brazil, Manaus – Amazonas, Reserva Florestal Adolfo Ducke	NY 00689602

Fig. S1 Androecium of Lecythidoideae. a, b, *Couratari asterotricha*. a, Overview of the fertile stamen with the background removed; the horizontal lines and numbering correspond to those in b. b, Cross-sections of the fertile anther. Note the idioblasts with phenolic compounds in the anther epidermis. c, d, e, f, *Corythophora alta*. c, Overview of the fertile stamen from the staminal ring with the background removed; the horizontal lines and numbering correspond to those in d. d, Cross-sections of the fertile anther. e, Overview of the staminode from the androecial hood with the background removed; the horizontal lines and numbering correspond to those in f. f, Cross-sections of the infertile anther. Scale bars: a, 500μm; b, e, 200μm; c–d, f, 100μm.

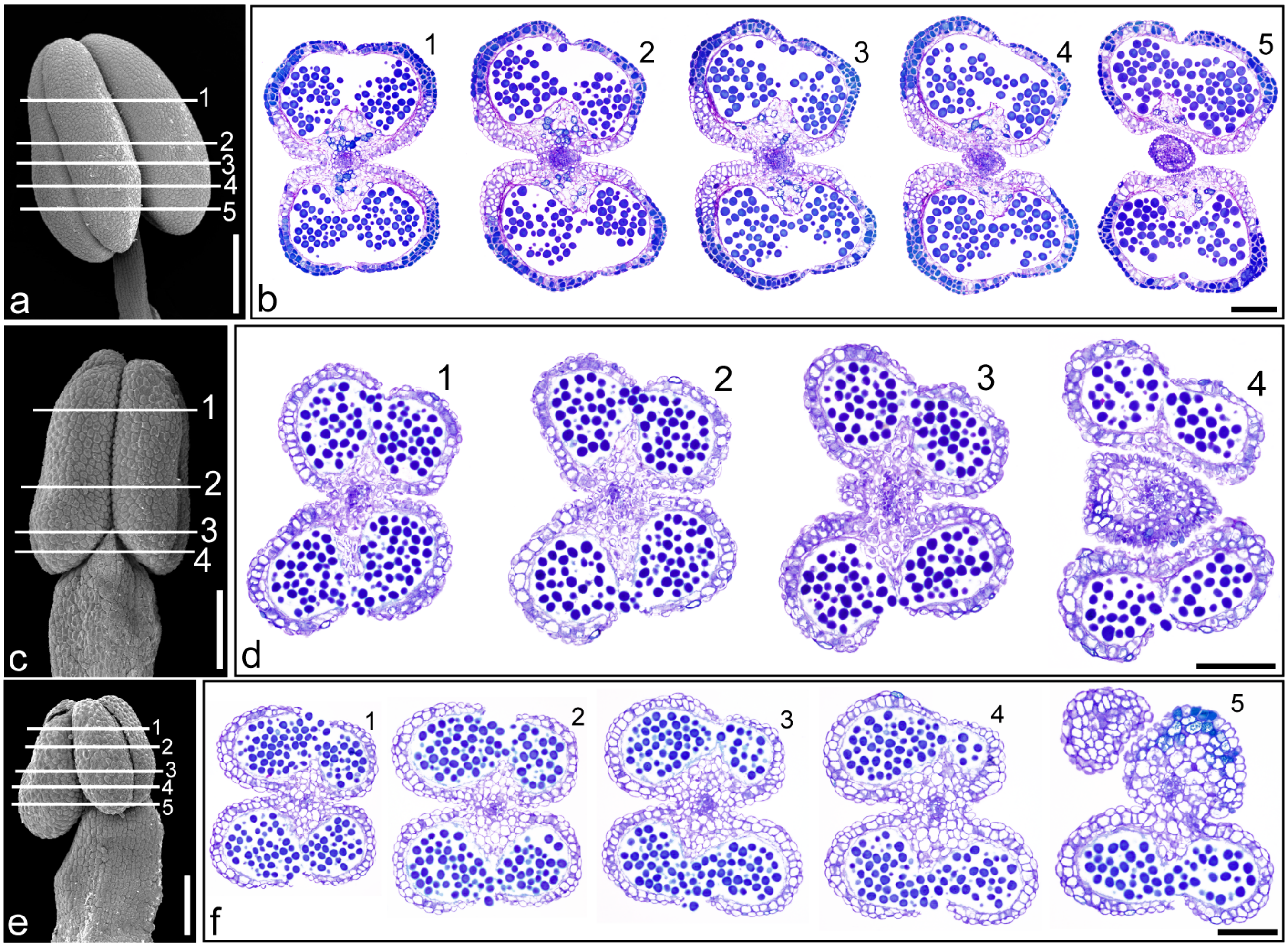

Fig. S2 Structural characterization of nectaries in species of the genus *Bertholletia* and *Lecythis* using light microscopy. a-d, *Bertholletia excelsa*. a, Longitudinal section of the inner region of the androecial hood with nectar-secreting staminodes. Note the course of the vascular bundle. b, Detail of the region of insertion of the staminode in the androecial hood, showing the peripheral vascular bundle. c, Cross-section of the base of the staminode showing the peripheral vascular bundle. d, Cross-section of the base of the staminode under fluorescence after staining with Nile Red, showing the discontinuity of the cuticle in the region of the vascular bundle. e-f, *Lecythis gracieana*. e, Longitudinal section of the inner region of the androecial hood with nectarsecreting staminodes. Note the course of the vascular bundle. f, Cross-section under autofluorescence of the base of the staminode showing the peripheral vascular bundle, (*) indicates nectar secretion. g-h, *Lecythis prancei*. g, Detail of the region of insertion of the staminode in the androecial hood, showing the peripheral vascular bundle, (*) indicates nectar secretion. h, Detail of the base of the staminode showing the peripheral vascular bundle and nectar secretion (*). i, *Lecythis poiteaui*. Cross-section of the infertile anther of a nectar-producing staminode, note that the esporoderm of the pollen grains is collapsed, indicating pollen inviability. Abbreviations: ah, androecial hood; ct, cuticle; ia, infertile anther; ns, nectariferous staminode; nt, nectariferous tissue; pg, pollen grain; sc, secretory cell; st, sieve tube; te, tracheal element; vb, vascular bundle. Scale bars: a, e, 200µm; c, i, 100µm; b, d, f-h, 50µm.

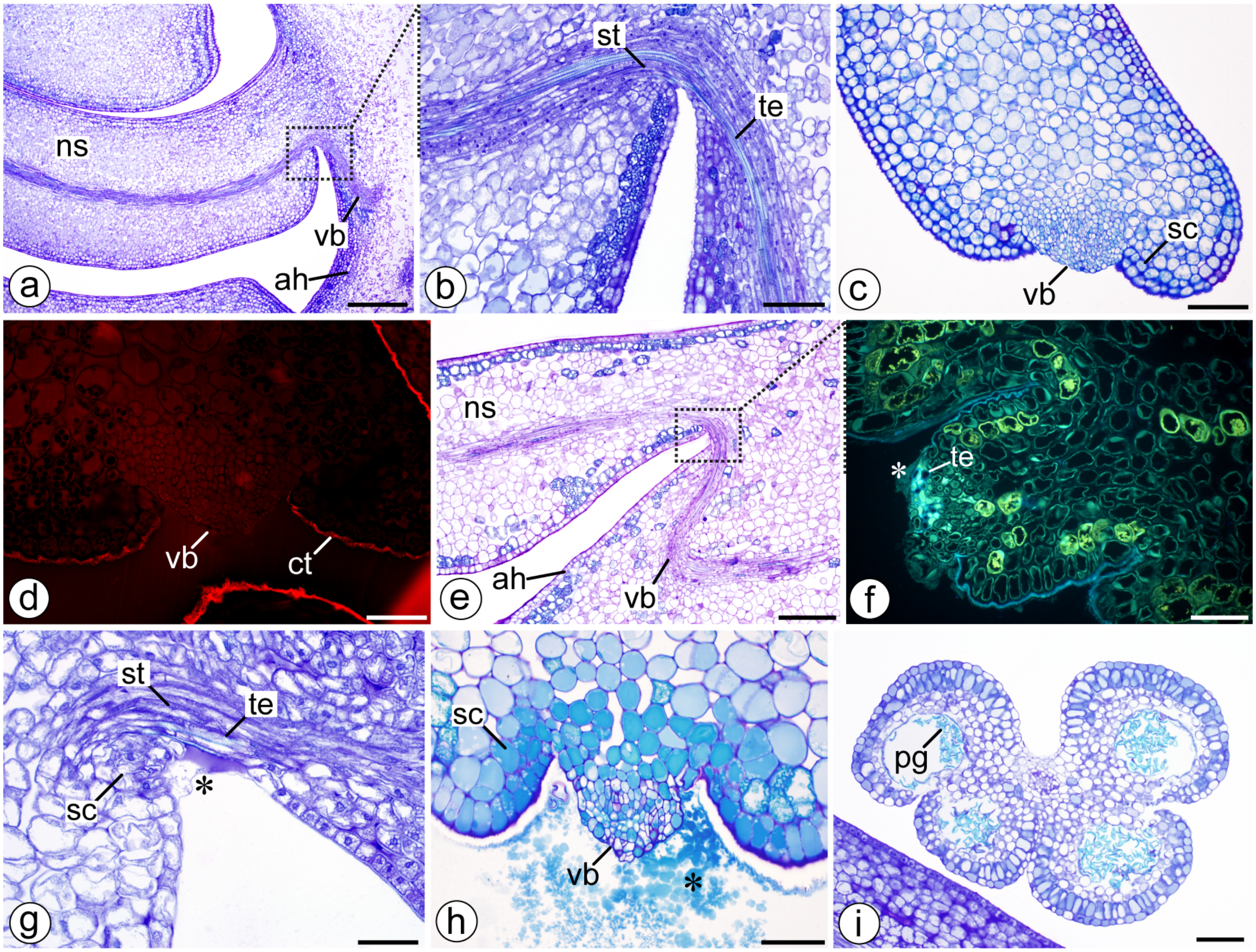
a
ns
vb
ah
b
st
te
c
sc
vb
d
vb
ct
e
ns
ah
vb
f
*
te
g
st
te
sc
*
h
sc
vb
*
i
pg

Fig. S3 Simmap of ancestral state estimations for kind of floral reward (left) and floral symmetry (right) on the best-scoring and time-calibrated tree of Lecythidoideae. Pie charts at the nodes indicate the posterior probabilities (<100%) of each state, based on 200 stochastic mappings.

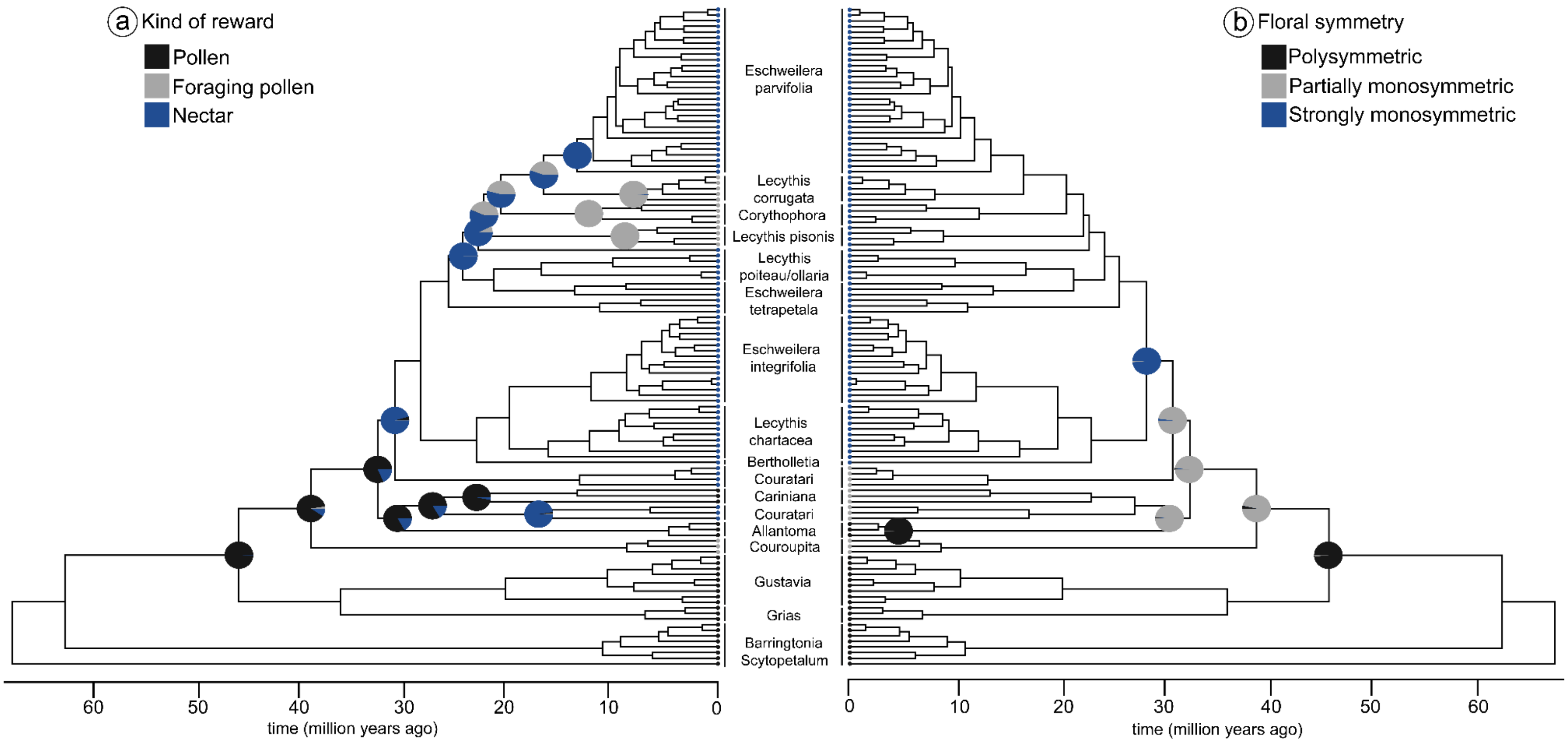

Table S2 Estimated number of transitions between floral rewards and floral symmetry in Lecythidoideae.

Trait	Ancestral state	Derived state	Number of Transitions
**Floral reward**			
	Nectar	Fertile pollen	0.61
	Nectar	Foraging pollen	2.28
	Fertile pollen	Nectar	1.97
	Fertile pollen	Foraging pollen	0.94
	Foraging pollen	Nectar	0.58
	Foraging pollen	Fertile pollen	0.06
**Floral symmetry**			
	Partially monosymmetric	Strongly monosymmetric	0.99
	Partially monosymmetric	Polysymmetric	1.01
	Strongly monosymmetry	Partially monosymmetric	0.02
	Strongly monosymmetry	Polysymmetric	0.02
	Polysymmetry	Partially monosymmetric	1.08
	Polysymmetric	Strongly monosymmetric	0.03
